# Insights into peculiar fungal LPMO family members holding a short C-terminal sequence reminiscent of phosphate binding motifs

**DOI:** 10.1038/s41598-023-38617-5

**Published:** 2023-07-18

**Authors:** Jean-Lou Reyre, Sacha Grisel, Mireille Haon, Ruite Xiang, Jean-Charles Gaillard, Jean Armengaud, Victor Guallar, Antoine Margeot, Simon Arragain, Jean-Guy Berrin, Bastien Bissaro

**Affiliations:** 1 UMR1163 Biodiversité et Biotechnologie Fongiques, INRAE, Aix Marseille University, 13009 Marseille, France; 2 IFP Energies nouvelles, 1 et 4 avenue de Bois-Préau, 92852 Rueil-Malmaison, France; 3 Barcelona Supercomputing Center, Plaça Eusebi Güell, 1-3, 08034 Barcelona, Spain; 4 ICREA, Passeig Lluís Companys 23, 08010 Barcelona, Spain; 5 Département Médicaments et Technologies pour la Santé (DMTS), SPI, Université Paris-Saclay, CEA, INRAE, 30200 Bagnols-Sur-Cèze, France; 6 INRAE, Aix Marseille University, 3PE Platform, 13009 Marseille, France

**Keywords:** Biochemistry, Biocatalysis, Carbohydrates, Enzymes

## Abstract

Lytic polysaccharide monooxygenases (LPMOs) are taxonomically widespread copper-enzymes boosting biopolymers conversion (e.g. cellulose, chitin) in Nature. White-rot Polyporales, which are major fungal wood decayers, may possess up to 60 LPMO-encoding genes belonging to the auxiliary activities family 9 (AA9). Yet, the functional relevance of such multiplicity remains to be uncovered. Previous comparative transcriptomic studies of six Polyporales fungi grown on cellulosic substrates had shown the overexpression of numerous AA9-encoding genes, including some holding a C-terminal domain of unknown function (“X282”). Here, after carrying out structural predictions and phylogenetic analyses, we selected and characterized six AA9-X282s with different C-term modularities and atypical features hitherto unreported. Unexpectedly, after screening a large array of conditions, these AA9-X282s showed only weak binding properties to cellulose, and low to no cellulolytic oxidative activity. Strikingly, proteomic analysis revealed the presence of multiple phosphorylated residues at the surface of these AA9-X282s, including a conserved residue next to the copper site. Further analyses focusing on a 9 residues glycine-rich C-term extension suggested that it could hold phosphate-binding properties. Our results question the involvement of these AA9 proteins in the degradation of plant cell wall and open new avenues as to the divergence of function of some AA9 members.

## Introduction

White-rot (WR) fungi are pivotal decomposers of dead organic matter in forest ecosystems that have developed various strategies to metabolize the plant biomass substratum, contributing thereby to the global carbon cycle^1^. To tackle the complexity of plant cell walls (PCW), a complex matrix composed of cellulose, hemicelluloses and lignin, a common strategy in the fungal kingdom consists in the secretion of a variety of enzymatic activities, notably carbohydrate-active enzymes (CAZymes) including hydrolases and oxidoreductases^2–4^. A clear picture of the scope of enzymatic activities involved and how these are interconnected and orchestrated remain a major gap in our understanding of biomass decay. A major step forward has been achieved in the past decade with the discovery of lytic polysaccharide monooxygenases (LPMOs)^5^, which has profoundly changed our vision of biomass conversion processes, at both fundamental and industrial levels^6–8^. Of note, in addition to these canonical activities on biomass, recent works have demonstrated the implication of some LPMOs in new divergent functions, such as microbial pathogenicity, cellular development or copper homeostasis^9^.

LPMOs are copper-dependent oxygenases catalyzing the oxidative cleavage of glycosidic bonds in various recalcitrant polysaccharides, including cellulose and chitin^5,10–13^. By doing so, LPMOs facilitate the depolymerization of polysaccharides by hydrolases, and thereby boost the overall conversion turnover. Today, LPMOs are classified as “auxiliary activities” (AA) within the CAZy database^14^, and further distributed into 8 families (AA9-AA11 and AA13-AA17). Among them, the AA9 family has been extensively studied for its ability to target and degrade cellulose, but also cello-oligosaccharides or hemicelluloses with glucan backbone^15–18^. To catalyze their reaction, LPMOs need a source of electron and an oxygenated substrate (O_2_ or H_2_O_2_). When fueled with H_2_O_2_, LPMOs catalyze an efficient peroxygenase reaction^19^, which entails the control of a powerful, and yet potentially harmful, Fenton-type chemistry^20^.

Remarkably, recent genome sequencing campaigns have highlighted the wide range of genes coding for LPMOs of the same family (especially AA9 enzymes, henceforth called AA9s), with up to > 60 different genes in some filamentous fungi^21^. The functional relevance of this redundancy is still not fully understood and the physiological role of most of these LPMOs remains unknown. Such LPMO-rich, white-rot filamentous fungi are notably found within the Polyporales order, belonging to the Agaricomycetes class and Basidiomycota division. Recently, Hage and colleagues assessed the functional adaptation of Polyporales to wood decay activities^22^ by performing a comparative transcriptional analysis of six Polyporales species (*Artolenzites elegans*, *Leiotrametes* sp*.*, *Pycnoporus cinnabarinus*, *Pycnoporus coccineus*, *Trametes ljubarskyi* and *Irpex lacteus*) grown on wheat-straw, pine, aspen and crystalline cellulose. When focusing on the transcriptional response of genes coding for AA9s, some LPMOs displaying an atypical, conserved C-terminal extension (called X282)^23^, and henceforth called AA9-X282, stood out. Interestingly, these AA9-X282s were all predicted as secreted and found among the most up-regulated LPMOs after 3 days of growth on cellulose^22^.

In this study, we investigated the role(s) of several of these atypical AA9-X282s. To this end, we produced six AA9-X282s in *Pichia pastoris*, displaying different modularities and carried out in-depth characterization using a combination of in silico and biochemical approaches, including enzymatic assays and substrate binding studies.

## Results

### AA9-X282s form a distinct clade among other AA9s

**Figure 1:**
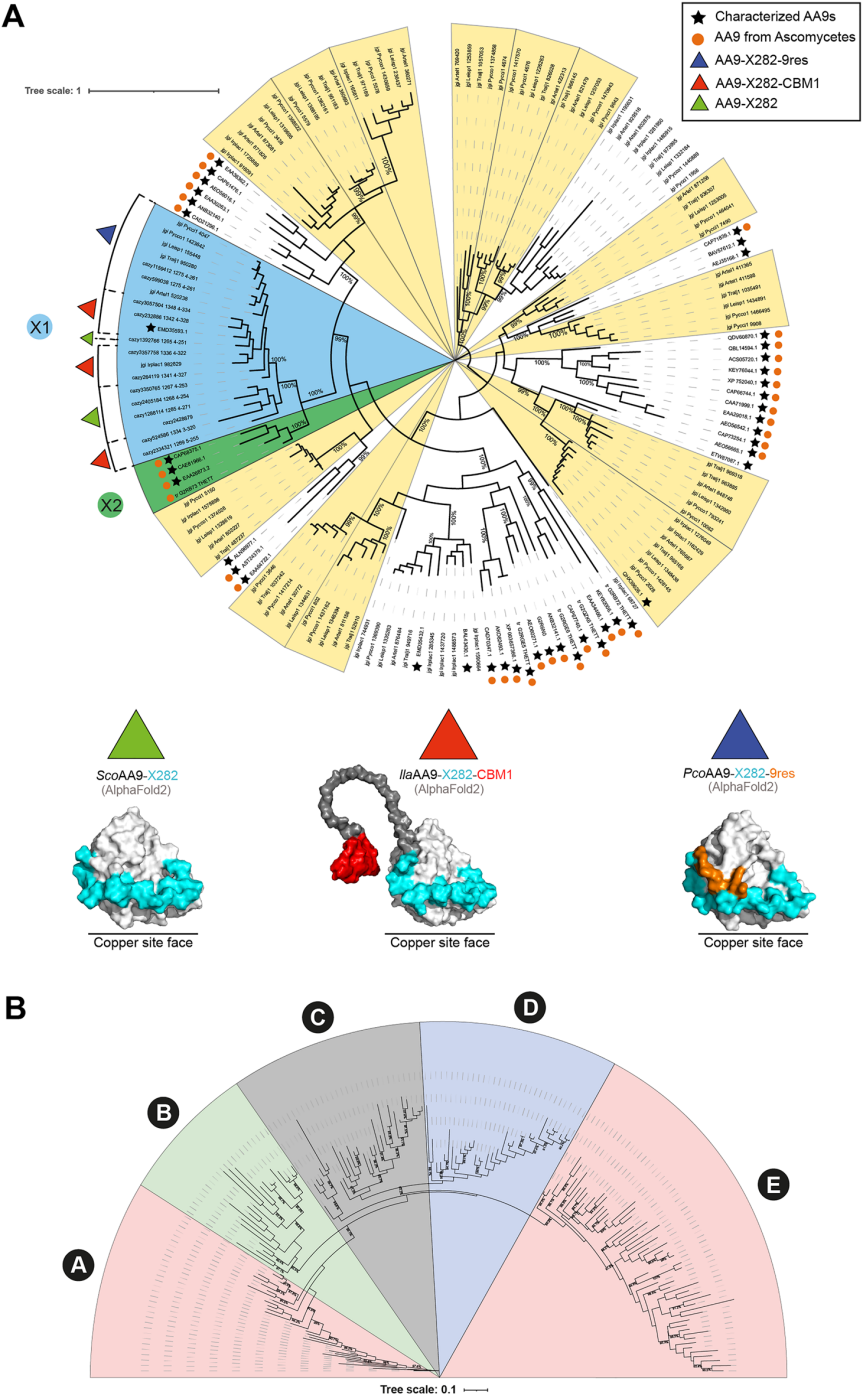
AA9-X282s within the phylogenetic tree of AA9s. (**A**) The tree was built with the catalytic domain only (i.e. without C-term extension, including the X282 extension) of 156 AA9 sequences including (i) 39 sequences of all the biochemically and/or structurally characterized AA9s available in the CAZy database (marked by a black star), (ii) 13 AA9-X282 sequences covering the diversity of their C-terminal modularity and (iii) 104 AA9s sequences regrouping all the AA9s from the six Polyporales studied by Hage et al.^22^. The tree was inferred using IQTree^28^ (1000 bootstraps; value displayed on tree as percentages) and visualized with Interactive Tree Of Life (ITOL) software^29^. Clades are colored in yellow when populated with AA9s from at least 5 out of the 6 *Trametes* species studied in Hage et al.^22^. Clade X1 contains AA9-X282s from Basidiomycetes and clade X2 contains AA9 orthologs (devoid of X282) found in Ascomycetes. The sequences from Ascomycetes are marked with an orange circle. The repartition of the AA9-X282 modularity is shown with colored triangles (see corresponding homology models of the three types of AA9-X282s at the bottom of panel A). In the structure predictions (AlphaFold2^30^), shown as surface, the catalytic domain is colored in light grey, the X282 extension in cyan, the 9res motif in orange, the linker in dark grey and the CBM1 in red. (**B**) Phylogenetic analysis of 174 AA9-X282 catalytic domains, showing 5 clades (A to E). Each clade is colored according to its most prevalent modularity (> 50% of sequences) using the same color code as in panel A (see Fig. S4 for more details). The tree was generated with IQ-Tree (1000 Bootstraps).

In the study by Hage et al., the AA9-X282s were found among the most up-regulated LPMOs after 3 days of growth of the six different Polyporales on cellulose (28–500-fold change in transcript read counts compared to the maltose control condition), and all clustered with cellulose-responsive genes (Fig. S1)^22^. Interestingly, only a single AA9-X282-encoding gene was identified for most Polyporales. Seeking for hints as to the potential activity of these AA9-X282s, we first performed a phylogenetic analysis to determine their evolutionary distance to other characterized AA9s (Fig. 1). To this end, we gathered the AA9 domain only of all the AA9s from the six Polyporales analyzed in Hage et al.^22^ (104 sequences) to which we added the 39 sequences of all the hitherto experimentally-characterized AA9s available in the CAZy database, and 13 other AA9-X282 sequences selected to fully cover the 3 different C-terminal modularities (see clade X1 in Fig. 1A), for a total of 156 sequences. Of note, X282-containing LPMOs are exclusively found in Basidiomycetes (and more precisely Agaricomycetes)^22^.

We show that AA9-X282s all cluster within a same clade, so-called clade X1 (Fig. 1A), indicating that the AA9 domain appended with a X282 extension has diverged from other LPMOs (average sequence identity of the AA9 domain of 44% with the closest “non-X282” AA9, Fig. S2). Noteworthy, this clade comprises only one characterized member, the AA9B from the Polyporales *Ceriporiopsis subvermispora* (syn. *Gelatoporia subvermispora*) (*Cs*LPMO9B), which harbors a X282 domain and a carbohydrate binding module from family 1 (CBM1) and has been shown to oxidize amorphous cellulose (i.e. phosphoric acid swollen cellulose; PASC) with strict C1-regioselectivity^24^. We underscore that in that study the reported oxidative activity (in terms of solubilized oxidized cello-oligosaccharides) seems rather low and that the presence of the X282 extension was not noted, and thus not specifically investigated.

Interestingly, the sister clade X2, which is devoid of X282 extension (Fig. 1A) but phylogenetically close to the clade X1 (percentage of identity ranging from 48 to 66%; Fig. S2) contains only AA9s from Ascomycetes fungi (in contrast to the Basidiomycete-specific clade X1). Three of these AA9s belonging to clade X2 were previously characterized: LPMO9B from *Podospora anserina* (*Pa*LPMO9B), C4-oxidizing on cellulose^25^; LPMO9J from *Neurospora crassa* (*Nc*LPMO9J), C1-oxidizing on cellulose^26^; and LPMO9E from *N. crassa* (*Nc*LPMO9E), C1-oxidizing on cellulose^27^. Despite the absence of X282 extension, the catalytic sites of these AA9s share some common features with AA9-X282s (see below, Fig. S3).

On a more general matter, the phylogenetic analysis also shows that the six Polyporales harboring an AA9-X282 also possess other AA9s, which tend to cluster in distinct clades (colored in yellow in Fig. 1A). These observations suggest that these white-rot fungi have evolved a set of distinct and conserved LPMOs that may fulfill different functions. We also underscore that AA9s from Ascomycetes do not cluster in a unique clade but are rather scattered across the tree (Fig. 1A), further supporting the idea that sequence divergence among AA9s would rather be due to neofunctionalization than to speciation.

### The X282 extension is found in three distinct modular architectures

After investigating the phylogenetic relationship of AA9-X282s to other AA9 family members, we carried out a deeper analysis of AA9s containing the X282 extension (174 sequences in the CAZy database). We identified three distinct C-term architectures based on the primary sequences: (i) AA9 harboring only a C-terminal X282 extension [AA9-X282]; (ii) AA9 possessing a CBM1 after the X282 extension [AA9-X282-Linker-CBM1]; and (iii) AA9 holding a conserved motif of 9 residues of unknown function (henceforth called “9res”), directly following the X282 extension [AA9-X282-9res] (Fig. 1A). Structural predictions (AlphaFold2)^30^ of a representative of each modularity are shown in Fig. 1A.

A phylogenetic analysis of the catalytic domain of the 174 AA9-X282 sequences available in the CAZy database allowed to identify five clades (Fig. 1B and Fig. S4). This analysis revealed that the three AA9-X282 modularities can be found in every clade and that AA9-X282-CBM1 populates mainly clade A and E and is the most represented modularity (56% of sequences), followed by the AA9-X282-9res (23%) and AA9-X282 (21%) (see Fig. S4 for more details). The presence and frequent occurrence of the CBM1 modularity is indicative of activity on PCW polysaccharides. The AA9-X282-9res and AA9-X282 modularities mainly cluster in clades B and D, accounting respectively for 77% and 66% of the sequences. The fact that a predominant modularity stands out for each of the clades could suggest that the AA9 domain has to some extent evolved in a modularity-dependent manner. It is also worth noting that the presence of the 9res motif is strongly associated with the *Trametes* genus.

### The X282 extension is highly conserved and displays a predicted belt-like structure

**Figure 2:**
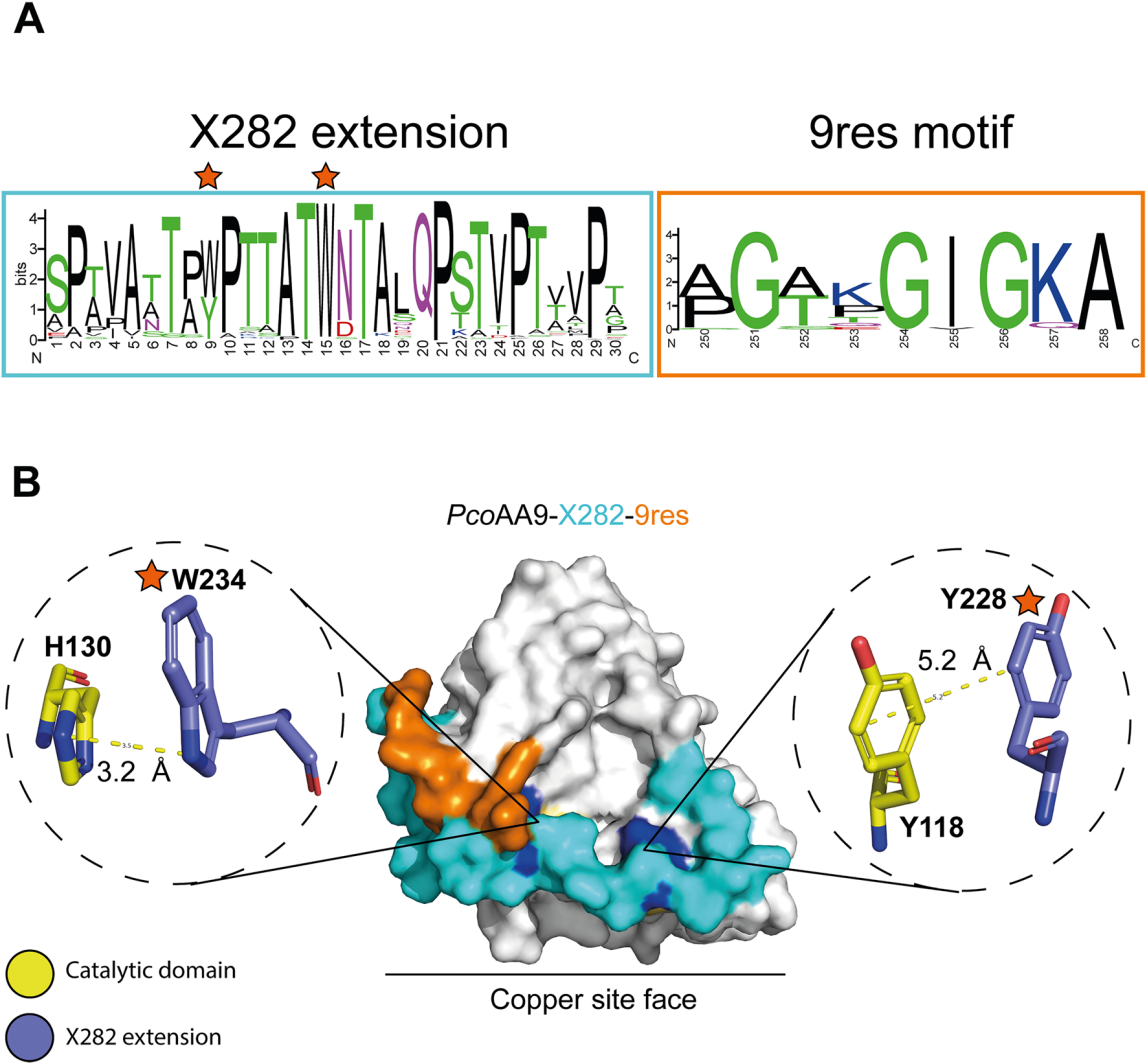
Structural analysis and amino acid conservation of the X282 extension. (**A**) WebLogo of the X282 extension and the 9res motif based on 174 sequences provided by the CAZy database. Conserved aromatic amino acids are marked with an orange star. (**B**) Homology model (AlphaFold2)^30^ of *Pco*AA9-X282-9res shown as surface. The X282 extension and 9res motif are colored in cyan and orange, respectively. Zoom-in views (in dotted circles) show the X282 aromatic amino acids (shown as blue sticks) potentially involved in π-stacking interactions with amino acids from the AA9 domain (shown as yellow sticks).

The X282 extension is a highly conserved stretch of approximately 30 amino acids (Fig. 2A). Its sequence is mostly made up of threonine/serine which could potentially serve as *O*-glycosylation or phosphorylation sites, while five conserved prolines could confer rigidity (Fig. 2A). Interestingly, two conserved aromatic amino acids (Trp/Tyr) are also present (marked by orange stars in Fig. 2). In CAZymes, such surface-exposed conserved aromatic residues are usually involved in substrate binding via CH-π stacking interactions^31^. When present in short modules they can be seen as hallmark of CBM modules^32^. Interestingly, all molecular models we generated for AA9-X282s rather predict π-stacking interactions with two highly conserved aromatic residues from the AA9 catalytic domain itself (Fig. 2B and Fig. S5). It is tempting to speculate that these interactions drive the X282 extension along the surface of the AA9 domain, hence forcing it to adopt a belt-like structure, the function of which is unknown.

Of note, we also observed another conserved feature in the AA9s from both X1 and X2 clades, namely a negatively charged patch (i.e. conserved acidic residues at the surface of the AA9 structure) next to the active site, just beneath the X282 extension (Fig. S3A). Laurent et al. demonstrated that phylogenetic clustering of AA9s is based on five extended catalytic site segments, annotated from Seg1 to Seg5^33^. We note that the sequence of this charged patch is part of segments 2 and 3 and diverge from other AA9s (Fig. S3B). Noteworthy, homology models suggest that these charged-patch sequences contain two strictly conserved cysteines facing each other, seemingly involved in a disulfide bond^33^ (Fig. S3B). The potential implication of these conserved features in a biological function also remains to be investigated.

### Biochemical characterization of the three AA9-X282 modularities

**Figure 3:**
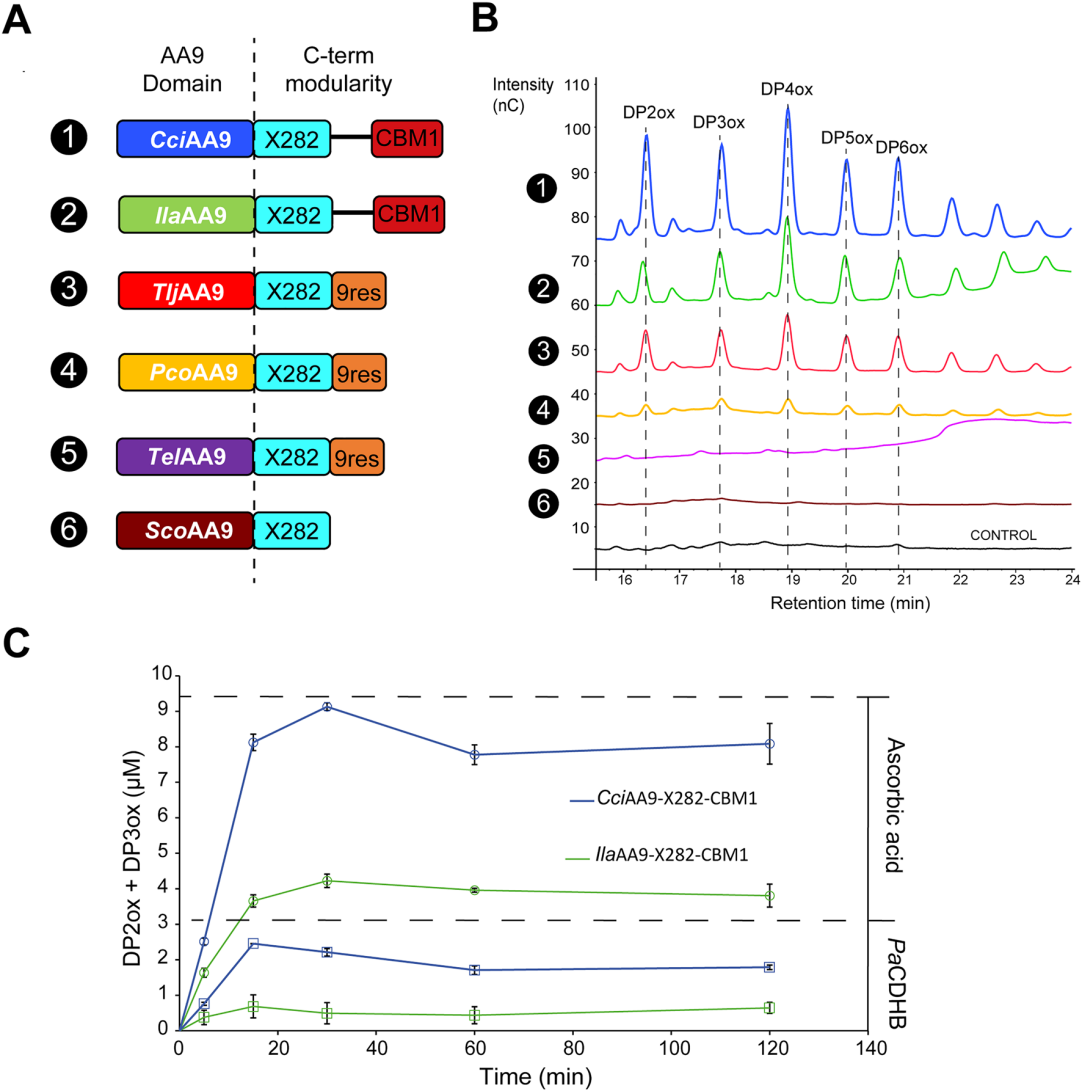
Kinetics and oxidative profile of the produced AA9-X282 on PASC. (**A**) Modularity of the studied AA9-X282s. Enzymes were named based on their organisms of origin: *Coprinopsis cinerea* (*Cci*), *Irpex lacteus* (*Ila*), *Trametes ljubarskyi* (*Tlj*), *Pycnoporus coccineus* (*Pco*), *Trametes elegans* (*Tel*), *Schizophyllum commune* (*Sco*). Each enzyme is associated with a color, consistently used in all panels. (**B**) HPAEC-PAD chromatogram of oxidized products released by the AA9-X282 (1 µM) from PASC (0.2%) in the presence of AscA (1 mM) after 24 h. In the control reaction the AA9 was replaced by CuSO_4_ (1 µM). (**C**) Time-course release of oxidized products (DP2ox + DP3ox) by *Cci*AA9-X282-CBM1 and *Ila*AA9-X282-CBM1 (1 µM each) from PASC (0.2%) using ascorbic acid (1 mM) or *Pa*CDHB (1 µM) as reductant. All reactions were incubated in sodium acetate buffer (50 mM, pH 5.2), in a thermomixer (850 rpm, 40 °C). Data points show average values and error bars show standard deviations (n = 3 independent biological replicates).

We heterologously produced in the yeast *Pichia pastoris* six recombinant AA9-X282 representatives of the three different modularities: (i) the AA9-X282-9res from *Pycnoporus coccineus* (syn. *Trametes coccinea*) (*Pco*AA9-X282-9res), *Trametes elegans* (*Tel*AA9-X282-9res) and *Trametes ljubarskyi* (*Tlj*AA9-X282-9res); (ii) the AA9-X282-CBM1 from *Irpex lacteus* (*Ila*AA9-X282-CBM1) and *Coprinopsis cinerea* (*Cci*AA9-X282-CBM1); and (iii) the AA9-X282 from *Schizophyllum commune* (*Sco*AA9-X282) (Fig. 3A). To assess the substrate specificity of these AA9-X282, we first assayed them on amorphous cellulose (PASC) (Fig. 3B). For some of the AA9-X282 LPMOs, we detected soluble C1-oxidized cello-oligosaccharides with degrees of polymerization (DP) between 2 and 6 (Fig. 3B). The most active ones harbored a CBM1. The intensity of the peaks observed for the AA9-X282-CBM1 (i.e. *Cci*AA9-X282-CBM1 and *Ila*AA9-X282-CBM1) are in good agreement with the ones observed for *Cs*LPMO9B, which also harbors a CBM1 and belongs to clade X1^24^. However, while keeping in mind potential variations due to equipment and buffers between different labs, we underscore that such intensities are very low when compared to other cellulose-active C1-oxidizing AA9-CBM1 LPMOs^17,34,35^ (Fig. S6A). Strikingly, most of the other AA9-X282 showed low (for *Pco*AA9-X282-9res) or no activity (*Tel*AA9-X282-9res and *Sco*AA9-X282) on cellulose. Therefore, we further screened a large range of potential PCW-derived substrates (including diverse xylans, mannans, pectins, cello-oligosaccharides and xylo-oligosaccharides; see Table S1), but no activity could be detected. To get deeper insights into the behavior of the two most active AA9-X282-CBM1, we carried out a time-course analysis (Fig. 3C). We observed a fast initial release of oxidized products from PASC, followed by early reaction ending, which is indicative of possible protein inactivation. Intriguingly, no synergy could be observed when using a CDH (*Pa*CDHB) as a redox partner, which is, to our knowledge, an unprecedented observation for AA9s (Fig. 3C). A control experiment using *Pa*AA9E LPMO illustrates how AA9s usually behave in the presence of CDH as redox partner and the expected amounts of products (Fig. S6). Intrigued by these observations, we evaluated whether the activity of the AA9-X282 was limited by other, usually key elements of an LPMO reaction, by notably testing the effect of (i) a broad range of reductants at two different pH (Fig. S7A, Table S1) and (ii) the addition of H_2_O_2_ as co-substrate (Fig. S7B). None of the tested conditions improved the activity of AA9-X282. Furthermore, we measured a rather low binding to PASC for the top 3 most active AA9-X282 (Fig. S7C), which is in agreement with the observed fast ending kinetic (Fig. 3C) since substrate binding has been shown to have a protective effect^19,36^ and this binding is improved by the presence of CBMs^37–39^. Despite poor activity on cellulose, all AA9-X282 LPMOs displayed an oxidase (i.e. H_2_O_2_ producing) activity (4–6 × 10^–3^ s^−1^) similar to that reported for other AA9s (10^–4^–10^–2^ s^−1^)^2^ (Fig. S7D).

In light of these results, and while bearing in mind their transcriptional upregulation and specific presence in wood-degrading fungi, we questioned the genuine biological role of these enzymes during PCW degradation. In search of new hints, we turned our attention towards the unusual structural features of these poorly active AA9-X282.

### The surface of *Cci*AA9-X282-CBM1, *Tlj*AA9-X282-9res and *Pco*AA9-X282-9res displays several phosphorylation sites

**Figure 4:**
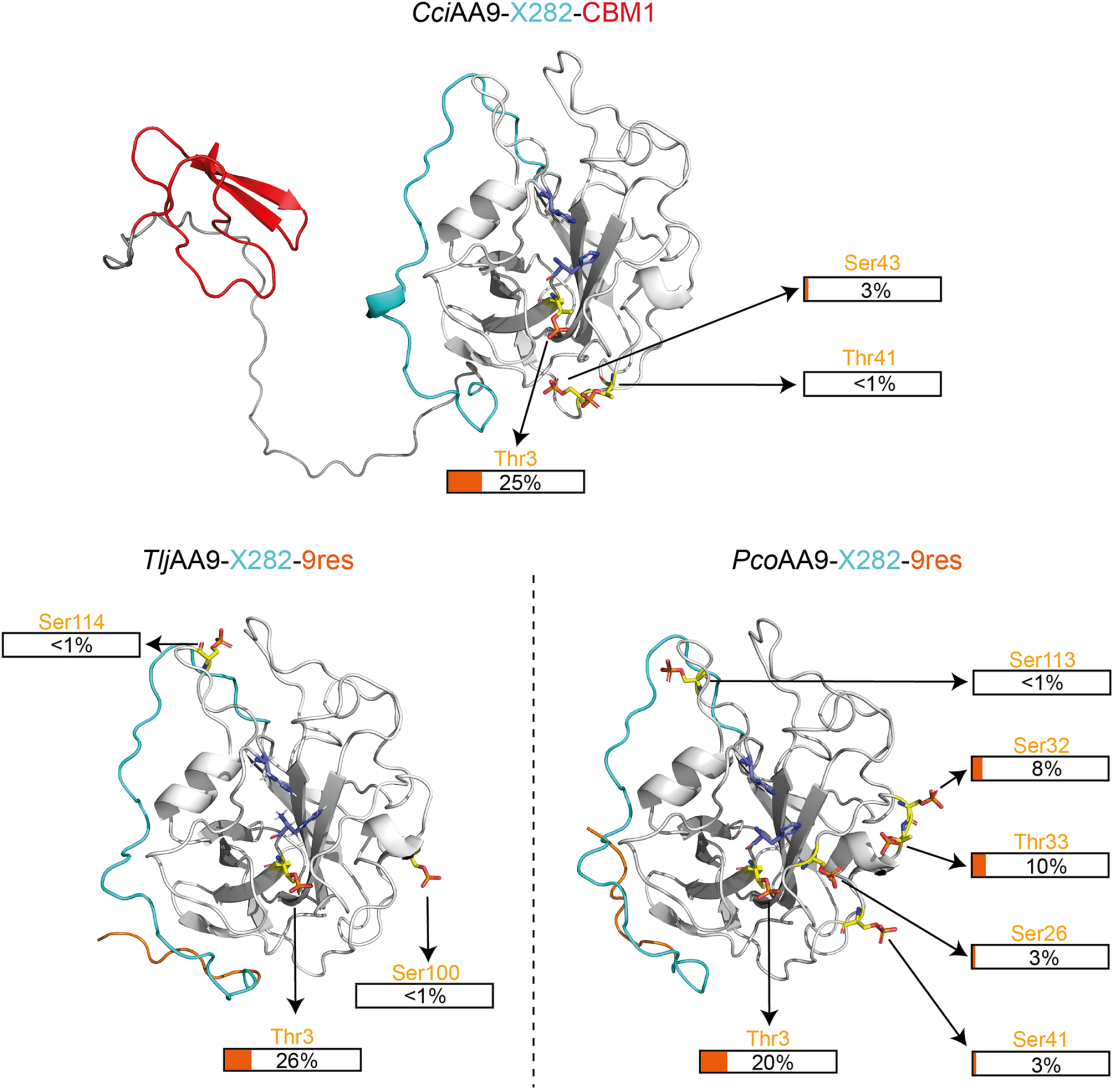
Mapping of phosphorylated residues on three AA9-X282 structure predictions. The phosphorylation (provided in the figure) of *Cci*AA9-X282-CBM1, *Tlj*AA9-X282-9res and *Pco*AA9-X282-9res were evidenced by tandem mass spectrometry. The identified phosphorylated residues are shown as yellow sticks. On the predicted protein structures (AlphaFold2^30^), shown as cartoon, the AA9 domain is coloured in white, the CBM1 in red, the X282 extension in cyan and the 9res motif in orange. The two catalytic histidines are shown as sticks and coloured in blue. Phosphate groups (shown as red sticks) have been added in PyMol using the PyTMs plugin. The percentages of peptides found phosphorylated compared to peptides found not phosphorylated is indicated for each position (we underscore that phosphorylated sites do not necessarily occur on a same protein molecule). The list of all peptides detected is provided in Table S2 (see 10.6084/m9.figshare.22709599).

When exploring the conservation of the residues constituting the surface of the AA9-X282 catalytic site, we surprisingly noted a high amount of conserved serine and threonine residues. More precisely, throughout our pool of 174 AA9-X282 sequences, 17 serines and 21 threonines display > 50% conservation (Fig. S8A). Among these residues, 2 serines and 7 threonines are part of the X282 extension. Such occurrence of serines and threonines on the surface of AA9-X282 is not inconsequential, as these residues are known to be potential sites of post-translational modifications, which may alter the enzyme activity/properties. In a recent study, a phosphorylated serine was identified in the crystal structure of an atypical AA9 from the Polyporales fungus *Lentinus similis.* This AA9 (*Ls*AA9B) is an LPMO-like protein that lacks the conserved histidine brace motif found in LPMOs and displays protein phosphorylation near the N-terminal residue^40^. Interestingly, structural alignment of *Pco*AA9-X282-9res with *Ls*AA9B (Fig. S8B) allowed us to identify a conserved serine (Ser24, 93% conservation) at the same position than the phosphorylated *Ls*AA9B-Ser25. On the basis of these observations, we decided to probe by high-resolution tandem mass spectrometry the phosphorylation state of three AA9-X282, namely *Cci*AA9-X282-CBM1, *Tlj*AA9-X282-9res and *Pco*AA9-X282-9res (Fig. 4 and Fig. S8A).

Strikingly, while none of the positions corresponding to Ser25 in *Ls*AA9B were observed phosphorylated, several other AA9-X282 Ser and Thr residues (two threonines and one serine for *Cci*A9-X282-CBM1; one threonine and two serines for *Tlj*AA9-X282-9res; two threonines and four serines for *Pco*AA9-X282-9res), all located at the surface of the proteins, were confidently proved to be phosphorylatable (Fig. 4). Intriguingly, the most documented site, with 20% to 26% of peptides covering this site being phosphorylated, was the threonine residue at position 3 (Thr3), which is conserved in almost all AA9-X282 sequences (99%) (Fig. S8A). We underscore that such observation at this position, replicated on several enzymes, is not fortuitous and highlights a possible regulation of the global charge of this patch of residues by kinases/phosphatases interplay. Considering the vicinity of the Thr3 with the N-term His1, it was tempting to speculate that this phosphorylation could affect the structural/redox properties of the histidine brace, which could in turn explain the low activity of these enzymes. To probe this hypothesis, we expressed in *Pichia pastoris* a mutated version (T3V) of the gene coding for *Pco*AA9-X282-9res, and after purification, tested its activity on PASC. The T3V mutation did not allow to restore any significant LPMO activity on cellulose (Fig. S9).

Altogether, these unexpected observations suggest the involvement of the AA9-X282 in a biological function potentially diverging from PCW degradation. Along this line, we focused our attention on the short C-terminal, conserved motif “9res” which displays unusual features.

### The 9-residues motif of AA9-X282s is reminiscent of canonical phosphate-binding motifs

**Figure 5:**
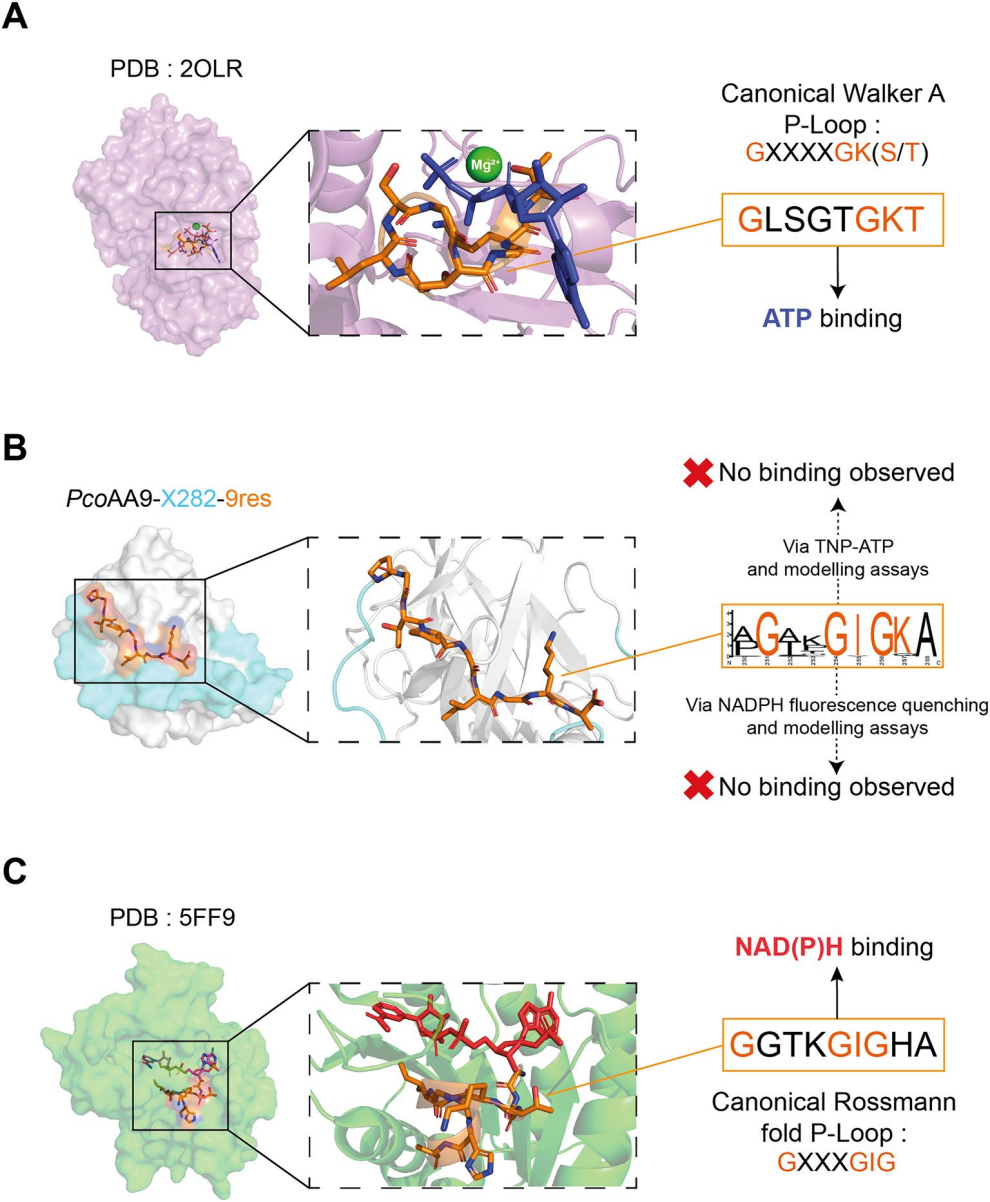
Structural comparison of the “9res” motif with canonical phosphate binding motifs. (**A**) ATP-dependent enzyme (PDB id 2OLR)^47^. In the canonical sequence “GXXXXGK(S/T)”, the lysine residue is crucial for the coordination of the nucleotide β- and γ-phosphates while the serine/threonine residue ensure the coordination a Mg^2+^-ATP complex required for its hydrolysis^41,48,49^. ATP is colored in blue and the Mg^2+^ cation is colored in green. (**B**) *Pco*AA9-X282-9res structure prediction (AlphaFold2) and zoom-in view of the 9res motif, colored in orange. (**C**) Zoom-in of the NADPH binding site of a canonical NADPH oxidase crystal structure (PDB id 5FF9)^50^. Key amino acids from the P-loop involved in phosphate binding are colored in orange. NADPH is colored in red.

The 9res motif is predominantly found in clades C and D in the tree of AA9-X282 proteins and is rather specific of the *Trametes* genus (Fig. S4). Indeed, we underscore that each *Trametes* species possess only one type of AA9-X282, which is the AA9-X282-9res, the only exception being *Trametes punicea*, that possesses both one AA9-X282-9res and one AA9-X282-CBM1. Looking more closely into this motif, we noted the presence of five strictly conserved residues (3 Gly, 1 Ile, and 1 Ala) and one highly conserved lysine (sometime replaced by a glutamine) (Fig. 2). The consensus sequence of this motif “GXXGIGKA” (where “X” can be any amino acids), together with the striking presence of phosphorylated residues in the protein, drew our attention due to its similarity with canonical and widespread phosphate binding loops (P-Loop) found in Walker A and Rossmann fold motifs^41,42^ (Fig. 5). The Walker A motif is a key feature in the AAA + (“ATPases associated with various cellular activities”) proteins superfamily for its ability to bind ATP (Fig. 5A)^43^. The Rossmann fold refers to a tertiary protein fold composed of a succession of β-strands connected by α-helices^44^ and that can accommodate dinucleotide substrates such as FAD, NAD or NADP^45^. One of the motifs specific to NAD(P)H, have been described as “GXXXGIG”^46^ (Fig. 5A).

To evaluate the potential of the 9res motif to bind ATP (Fig. S10) or NADPH (Fig. S11), we used both fluorometric and in silico approaches. Of note, to avoid any biases coming from C-term modifications, we cloned, produced, and purified a non-tagged version of *Pco*AA9-X282-9res devoid of C-terminal His-tag (referred to as “*Pco*AA9-X282-9res NT”). Nevertheless, both the non-tagged and His-tagged variants were evaluated. In the tested conditions, in vitro assays did not allow to show any significant ATP binding (Fig. S10A) nor ATP hydrolysis (Fig. S10B), and no significant binding to NADPH (Fig. S11A,B). In agreement, PELE site finder simulations did not identify the 9res motif as a suitable binding site for ATP (Fig. S10C) or NADPH (Fig. S11C), as the energetic minima, indicative of the location for putative binding sites, are far from the lysine of the "GXXGIGKA" consensus sequence for both ligands. This contrasts with the site finder simulations in a reference ATPase model (PDB id 1XU4), where, as expected, we observed a clear minimum in proximity to the lysine of the Walker A motif when simulated with ATP (Fig. S10C). However, it may be worth noting that, in the case of NADPH, albeit interaction energies remain low, a cluster with a relatively low distance between *Pco*AA9-X282-9res and NADPH was observed (Fig. S11C). It is plausible that the variations observed in the 9res motif of *Pco*AA9-X282-9res, compared to canonical Walker A and Rossman fold, may alter hypothetical binding to substrates and, in such alternative, related substrates remain to be found and tested. The conservation and similarity between the 9res motif and canonical phosphate binding motifs remain remarkable and would deserve further study in the future.

## Discussion

Our initial objective was to identify and characterize efficient LPMOs for plant biomass degradation. Building on the published observation that some atypical AA9-encoding genes were co-regulated with cellulases during biomass decay, we set off to characterize several of these AA9s displaying a module of unknown function (i.e., X282). However, despite our efforts, we only observed very low LPMO-type activity (on cellulose), and even more striking was the absence of synergy with CDH. In that regard, we hypothesize that the X282 belt may hamper protein–protein interactions. Overall, this poor activity, regardless of the reaction conditions, seems to distance AA9-X282 from a straightforward polymer degradation context. We even wonder whether these AA9s should be referred to as LPMOs, although they display several hallmarks of genuine LPMOs such as a functional histidine brace (as shown by normal H_2_O_2_ producing activity) and the occurrence of a CBM1 in some members. Nevertheless, these results and observations forced us to take a step back while investigating further the atypical features of these AA9s.

At first glance, we had suspected that the X282 extension could correspond to a new CBM but bioinformatic analyses and structures predictions rather suggest that the X282 extension would cap the AA9 domain as a belt by means of two conserved π-stacking interactions involving strictly conserved aromatic residues. In this configuration, the X282 extension is found relatively close to the copper face, just on top of another conserved negatively charged patch. Noteworthy, the fact that the X282 extension can be found alone at the C-terminus or followed by two distinct modularities (i.e., the 9res motif or a linker-CBM1), suggests that the function the X282 extension and the other motifs are not specifically correlated. Interestingly, peptidic belts have been reported to increase thermostability in fungal glucoamylases^51^, and one may wonder if the X282 could fulfill a similar protective function in AA9-X282s.

The second conserved pattern found in some of the AA9-X282s, notably in those produced by fungi belonging to the *Trametes* genus, is a stretch of 9 residues referred to as “9res” motif. We observed that its conserved sequence appears surprisingly reminiscent of canonical P-loops found in ATP and NAD(P)^+^/NAD(P)H dependent enzymes. The latter proteins are described as involved in intracellular mechanisms and, to the best of our knowledge, such P-loop-like motif has never been reported for secreted enzymes. Nonetheless, the presence of extracellular ATP (eATP) has already been demonstrated to be involved in different types of interactions, such as in plant response to biotic and abiotic stresses^52^, or in fungi-bacteria interactions^53^. The exact role of eATP in these interactions is still poorly understood, and, although no significant binding to ATP could be observed in our conditions, its potential involvement in AA9-X282 functionality should not be definitely ruled out.

The third intriguing observation on AA9-X282 was brought by proteomic analyses, viz*.* the presence of multiple phosphorylated sites at the surface of the proteins. The presence of these phosphorylated residues could locally impact the charge of the protein and thus enable/prevent interactions with a substrate or protein. Assessing the global occupancy rate of phosphorylated sites per proteins, could allow to estimate the actual localization and occurrence of these potential domains of interaction.

We underscore that the present study stands as the second observation of a phosphorylated AA9 originating from a Polyporales fungus, as a single phosphorylated site has already been observed in the crystal structure of another AA9 from the polypore *Lentinus similis* (*Ls*AA9B)^40^. It is worth noting that so far, no correlation can be established between the phosphorylation of these AA9s and the absence/presence of LPMO activity. Indeed, *Ls*AA9B is devoid of any copper coordination site, and in the case of AA9-X282, the non-phosphorylated T3V mutant did not permit to restore any activity on cellulose. Phosphorylation of extracellular proteins has already been observed in the LPMO field: the virulence factor CbpD from the human pathogen *Pseudomonas aeruginosa* was shown to display 11 phosphorylated residues at its surface when secreted by the bacterium^54^, two of these residues being found on the chitin-binding surface^55^. While CbpD recombinantly produced in *E. coli* was demonstrated to be an AA10 LPMO with chitin-oxidizing activity^56^, the in vivo functional impact of phosphorylations remains to be studied. Given the well-documented importance of protein phosphorylation in various signaling/regulation processes^57,58^, our study, as well as the few other reports cited above, collectively prompt a thorough examination of the post-translational modifications occurring in LPMOs and an investigation of their biological importance.

## Conclusions

Overall, we have shown that the AA9-X282 proteins display several unique features that have never been reported in the LPMO field. We believe that the co-occurrence of structural features reminiscent of phosphate binding loops, together with surface-exposed phosphorylation sites are not the fruit of fortuitous evolution and may be pieces of a larger puzzle. Recent work suggests that the functionality of LPMOs goes beyond their usually ascribed function of recalcitrant polysaccharide oxidizers^9,59^, such as in some pathogenicity mechanisms^56,59^, or in allorecognition upon fungal cell–cell contact^60^ where the LPMO catalytic function is not always decisive. Given the wide expansion of AA9 genes in the genomes of some white rot fungi (sometime over 60 genes), it is tempting to hypothesize that, during millions of years of evolution, a certain extent of functional divergence has occurred. Importantly, we wish to stress that the majority of studies have focused on the N-terminal part of LPMOs (mainly on catalytic domain appended or not with a CBM). However, a large proportion of LPMOs contain C-terminal disordered regions^61^, modules of unknown functions or small extensions, which are usually removed to ease recombinant production and characterization. Alike the module-walking strategy that has allowed to identify new LPMO families by looking for the presence of well-known CBMs, we believe that further examination of the C-terminal regions and their unusual properties may be one of the keys to unveil new functions of these moonlighting and enigmatic proteins.

## Material and methods

### Materials

Most chemicals, including Avicel, lignin-derived compounds and horseradish peroxidase were purchased from Sigma-Aldrich (Saint-Louis, Missouri, United-states). TNP-ATP was purchased from Jena Bioscience (Jena, Germany). Oligo- and polysaccharides substrates were purchased from Megazyme (Wicklow, Ireland). Both α- and β-chitin were purchased from Mahtani Chitosan (Gujarat, India). PASC was prepared as described in Wood^62^.

### Enzymes cloning and production

The proteins were produced using the in-house 3PE platform (*Pichia pastoris* Protein Express; www.platform3pe.com) as described in Haon et al.^63^. The nucleotide sequence of the 6 AA9-X282 from *Coprinopsis cinerea* (*Cci*AA9-X282-CBM1, Uniprot ID: A8NCG7), *Irpex lacteus* (*Ila*AA9-X282-CBM1; GenBank: KAI0763186.1), *Pycnoporus coccineus* (*Pco*AA9-X282-9res; GenBank: OSD05161.1), *Trametes ljubarskyi* (*Tlj*AA9-X282-9res; GenBank: KAI0367364.1), *Trametes elegans* (*Tel*AA9-X282-9res; GenBank: KAI0761258.1) and *Schizophyllum commune* (*Sco*AA9-X282; GenBank: KAI4528925.1.) were codon optimized for expression in *Pichia pastoris*. Gene synthesis was performed by Genewiz (South Plainfield, New-Jersey, USA), the region corresponding to the native signal sequence was kept and the genes were inserted into the expression vector pPICZαA (Invitrogen, Cergy-Pontoise, France) in frame with a C-terminal poly-histidine tag, except for *Pco*AA9-X282-9res NT where the native C-terminal sequence remained unmodified. PmeI-linearized pPICZαA recombinant plasmids were used to transform competent *P. pastoris* cells by electroporation. Zeocin-resistant transformants were then screened for protein production. The SuperMan5 strain was used to produce *Sco*AA9-X282 and *Tel*AA9-X282-9res while the X33 strain was used for *Ila*AA9-X282-CBM1, *Cci*AA9-X282-CBM1, *Tlj*AA9-X282-9res, *Pco*AA9-X282-9res, *Pco*AA9-X282-9res NT and *Pco*AA9-X282-9res T3V. The best-producing transformants were grown in 2 L of BMGY media in flasks at 30 °C in an orbital shaker (200 rpm) for 16 h to an OD600 of 2 to 6. Expression was induced by transferring cells into 400 mL of BMMY media at 20 °C in an orbital shaker (200 rpm) for another 3 days. Each day, the medium was supplemented with 3% (v/v) methanol. The cells were harvested by centrifugation, and just before purification the pH of the supernatant was adjusted to 7.8 by addition of Tris–HCl buffer (1 M, pH 8) and was filtered on a 0.45 µm membrane (Millipore, Burlington, Massachusetts, USA).

### Protein purification

For the His-tagged proteins, the filtered culture supernatant was loaded onto a 5 mL HisTrap excel column (GE Healthcare, Bus, France) equilibrated with buffer A (Tris–HCl 50 mM pH 7.8, NaCl 150 mM, imidazole 10 mM) that was connected to an Äkta purifier 100 (GE Healthcare). His-tagged recombinant proteins were eluted with 50% of buffer B (Tris–HCl 50 mM pH 7.8, NaCl 150 mM, imidazole 500 mM). For *Pco*AA9-X282-9res produced without His-tag (so called “*Pco*AA9-X282-9res NT”), the protein was purified using a monoQ™ 10/100 GL anion exchange column (Cytiva; Freiburg im Breisgau, Germany) and eluted using a gradient of Tris–HCl buffer (20 mM, pH 8.0) containing 1 M NaCl. The peaks obtained after elution were loaded onto 10% Tris–glycine precast SDS-PAGE gels (BioRad), stained with Coomassie Blue. The fractions containing pure recombinant proteins were pooled together, concentrated and dialyzed against Tris–HCl buffer (20 mM, pH 8.0). The protein concentrations were determined by absorption at 280 nm using a Nanodrop NB-2000 spectrophotometer (Thermo Fisher Scientific, Waltham, MA, USA). The protein parameters were the following: *Cci*AA9-X282-CBM1 (MW = 35,346.57 g.mol^−1^, ε280 = 63,995 M^−1^.cm^−1^); *Ila*AA9-X282-CBM1 (MW = 33,739.25 g.mol^−1^, ε280 = 58,495 M^−1^.cm^−1^); *Pco*AA9-X282-9res (MW = 27,694.72 g.mol^−1^, ε280 = 40,255 M^−1^.cm^−1^); *Pco*AA9-X282-9res-T3V (MW = 27,692.75 g.mol^−1^, ε280 = 40,255 M^−1^.cm^−1^); *Pco*AA9-X282-9res NT (MW = 26,871.88 g.mol^−1^, ε280 = 40,255 M^−1^.cm^−1^); *Tlj*AA9-X282-9res (MW = 27,732.86 g.mol^−1^, ε280 = 37,275 M^−1^.cm^−1^); *Tel*AA9-X282-9res (MW = 27,638.74 g.mol^−1^, ε280 = 38,765 M^−1^.cm^−1^); *Sco*AA9-X282 (MW = 26,829.63 g.mol^−1^, ε280 = 45,755 M^−1^.cm^−1^).

### Phylogenetic analysis

To build the phylogenetic tree of AA9s, we used 156 AA9 sequences including 39 sequences of all the biochemically and/or structurally characterized AA9s available in the CAZy database at the time of this study, 13 AA9-X282 sequences covering the diversity of their C-terminal modularity and 104 AA9s sequences regrouping all the AA9s from the six Polyporales studied by Hage et al.^22^. Of note, the variable C-terminal regions of all AA9 sequences were cut using BioEdit^64^ in order to keep the catalytic domain only. The 156 AA9 sequences batches were aligned using MAFFT-DASH (L-INS-i method)^65^, which include structural data input. The resulting multiple sequence alignments were used to infer the phylogenetic trees via the IQ-tree webserver. To infer the tree, the Whelan and Goldman (WAG) amino acid substitution model was selected with a maximum Likelihood optimized (ML-optimized) state frequency. Branch support was calculated by 1,000 bootstraps alignments using the ultrafast bootstrap analysis method. To build the phylogenetic tree of AA9-X282 proteins only, 174 sequences available in the CAZy database were retrieved and the same procedure as above was followed.

### LPMO activity assays

Substrates screening of AA9-X282s were performed on 16 substrates (Table S1). All substrates were tested at a concentration of 5 mM (if soluble in water) or 5 mg.mL^−1^ (if insoluble in water). Reaction on PASC were performed at a concentration of 2 mg.mL^−1^ (eq. 0.2% w/v), and PASC/Xylan (from beechwood) coupling were tested in different proportions (0.4% PASC /0.4 or 0.3 or 0.2 or 0.1 or 0.04% xylan; % in w/v). All reactions were performed in sodium acetate buffer (50 mM, pH 5.2) in a final volume of 500 µL and incubated for 24 h, unless stated otherwise (30 °C, 850 rpm). Reactions were stopped by heating at 100 °C for 10 min and then centrifugated before analysis of the soluble fraction using a high-performance anion-exchange chromatography (HPAEC) coupled with pulsed amperometric detection (PAD) (DIONEX ICS6000 system, Thermo Fisher Scientific). The system is equipped with a CarboPac-PA1 guard column (2 × 50 mm) and a CarboPac-PA1 column (2 × 250 mm) kept at 30 °C. Elution was carried out at a flow rate of 0.25 mL.min^−1^ and 25 µL of sample was injected. The solvents used were 100 mM NaOH (eluent A) and NaAc (1 M) in 100 mM NaOH (eluent B), and the following gradient was applied: 0 to 10 min, 0 to 10% B; 10 to 35 min, 10 to 35% B (linear gradient); 35 to 40 min, 30 to 100% B (curve 6); 40 to 41 min, 100 to 0% B; 41 to 50 min, 100% A. Integration was performed using the Chromeleon 7.2.10 chromatography data software.

### AA9-X282s reductants screening

14 lignin-derived compounds were tested as reductants for *Ila*AA9-X282. Reaction on PASC (0.2%) were performed as described above at pH 5.2 (50 mM sodium acetate buffer) or pH 8.0 (50 mM Tris–HCl buffer) using 1 mM of reductant and stopped by heating 10 min at 100 °C. After cooling down, the pH 8 reactions were brought back to pH 5–6 by adding 25 μL of acetic acid (1 M). A cellulolytic cocktail from the Ascomycete *T. reesei* containing two cellobiose hydrolases, two *endo*-glucanases and one β-glucosidase was then added to all reaction broths at a total concentration of 100 mg/g of substrate and complete hydrolysis of PASC was ensure by incubating the reactions for 24 h in a Thermomixer (50 °C, 850 rpm). The complete fractions (soluble and insoluble) of oxidized products (DP2ox and DP3ox) were then quantified by HPAEC-PAD, using a standard curve.

### AA9-X282s oxidase activity assays

Oxidase activity of the six AA9-X282s was measured according to the protocol described by Kittl et al.^26^. The reaction mixture contained Amplex Red reagent (200 µM; Thermo Fisher Scientific), Horseradish peroxidase (HRP; 0.1 mg.mL^-1^ final) and AA9-X282s (1 µM). The reactions were performed in 96-well microplates in sodium phosphate buffer (50 mM, pH 7.0) and started by adding 50 μM of ascorbic acid. The H_2_O_2_ production rate is reflected by the production rate of resorufin, i.e. the product of Amplex Red oxidation by HRP and H_2_O_2_, which was monitored at 575 nm over a 30 min period. The initial slope of the curve, normalized with the enzyme concentration, was used to calculate the H_2_O_2_ production rate.

### AA9-X282 cellulose binding assays

The binding affinity of AA9-X282s (1 µM) for PASC (0.2%) and Avicel (5 mg.mL^−1^) was monitored by measuring the concentration of the protein in the supernatant after 2 or 24 h of incubation in a Thermomixer (30 °C, 850 rpm). For each condition, a control reaction in the absence of substrate was carried out. The protein concentration was measured at 280 nm using a Nanodrop ND-2000 spectrophotometer (Thermo Fisher Scientific, Waltham, MA, USA). A binding of 0% corresponds to the enzyme absorbance in the absence of substrate. All the reactions were performed at pH 5.2 (50 mM sodium acetate buffer) or pH 7.0 (50 mM sodium phosphate buffer). Only the results at 2 h are shown since no increase in binding could be observed after 24 h of incubation.

### Tandem mass spectrometry analysis of AA9-X282s

Protein samples (20 µg) were diluted into 200 µL of trifluoroacetic acid (0.1%) and desalted with C18 spin columns (Harvard Bioscience). After their elution, polypeptides were dried and dissolved into 20 µL of 100 mM NH_4_HCO_3_. Four volumes of 5 µL corresponding to 5 µg of protein were treated for 10 min with 5 mM dithiothreitol at 56 °C under gentle agitation, subjected to 5 mM iodoacetamide for 10 min at room temperature in the dark, and then proteolyzed for 2 h by either trypsin, AspN, trypsin and AspN, trypsin and GluC proteases prepared at 0.1 µg per µL and used at 2% of protein biomass. After proteolysis, trifluoroacetic acid was added (0.1% final volume). The resulting peptides (200 ng) were resolved by reverse phase chromatography on a Vanquish Neo UHPLC (Thermo) coupled to an Exploris 480 tandem mass spectrometer (Thermo). This instrument was operated in data-dependent acquisition mode as previously described^66^. The gradient applied on the reverse phase column was developed over 30 min from 4 to 40% of acetonitrile in 0.1% of formic acid. MS full scan spectra and MS/MS spectra after fragmentation were acquired at resolution 120,000 and 60,000, respectively. Only ions with 2 or 3 positive charges were selected for fragmentation with a 10-s dynamic exclusion window. MS/MS spectra analysis was performed as previously described^67^ with carboxyamidomethylation of cysteine residues as fixed modification, oxidation of methionine, deamidation of asparagine and glutamine, and phosphorylation of serine and threonine as variable modifications. For each residue, the phosphorylation rates were estimated by dividing the number of MS/MS spectra assigned to peptides containing a phosphorylation by the total number of MS/MS spectra attributed to peptides encompassing the phosphorylatable residue. We underscore that such percentage does not represent the true proportion of phosphorylated proteins in each sample as phosphorylated and unphosphorylated peptides may ionized differently in our analytical conditions.

### ATP binding assays

Fluorescent emission scanning spectra of TNP-ATP were obtained using a Biotek Synergy HT plate-reader (Bio-Tek Instruments, Winooski, VT, USA), equipped with emission and excitation filters of 540 (± 20) nm or 410 (+ /− 20) nm respectively. Binding assays were performed by mixing 10 µM of enzyme with 20 µM of TNP-ATP in Tris–HCl buffer (50 mM, pH 7.5) and measuring the change in TNP-ATP fluorescence emission at 540 nm upon excitation at 410 nm. All measurements were carried out in 96-well, black, flat-bottom plates (Corning) at 25 °C, in 100 μL reaction volumes.

### NADPH binding assays

The binding of *Pco*AA9-X282-9res and *Pco*AA9-X282-9res NT to NADPH was monitored based on a method described in Fjeld et al.^68^. Proteins were excited at 285 nm and fluorescence emission was measured at 325 nm (+ /− 20 nm). When exciting the mixture of NADPH and AA9-X282s at 285 nm, a conserved tryptophan (Trp234) present at the surface of AA9-X282s in the vicinity of the 9res motif is expected to transfer its emitted fluorescence at 325 nm to a potentially bound NADPH via a FRET mechanism. The resulting loss in fluorescence at 325 nm was monitored in a spectrofluorometer (Biotek Synergy HT plate-reader). To prevent any redox interactions between NADPH and the AA9-X282s that could modify the intrinsic fluorescence of the proteins as described elsewhere^69^, *apo*-AA9-X282s were prepared as described by Askarian et al.^56^. Briefly, *Pco*AA9-X282-9res and *Pco*AA9-X282-9res NT solutions (10 µM final) were incubated overnight with EDTA (2 mM) in Tris–HCl buffer (20 mM, pH 7.5) at 4 °C, followed by addition of MgCl_2_ (5 mM), further incubated during 15 min at room temperature, and finally desalted on a PD MidiTrap G-25 column. All fluorescence measurements were performed in 96-well, black, flat-bottom plates at 25 °C, in 100 μL reaction volumes.

### Modelling

PELE (Protein Energy Landscape Exploration) is a Monte Carlo-based simulation algorithm consisting of two stages. In the first stage, the ligand is randomly translated and/or rotated, and the protein is perturbed using a normal mode method based on an anisotropic network model (ANM). In the second stage, the side chains around the ligand are optimized using a rotamer library followed by an overall structural minimization. The new conformation is then accepted or rejected based on the Metropolis criterion^70^. Many protocols have been implemented using this algorithm to tackle different problems of biological interest. One of these protocols is Site Finder^71,72^ which performs a global exploration around the entire surface of the protein to find a suitable binding site for a given ligand. This is achieved by creating N copies of the structure, as many as the number of processors, where the ligand is randomly placed around the protein and allowed to run for a predefined number of PELE steps. The conformations with the lowest interaction energies are then refined with PELE by constraining the ligand to a local exploration in order to generate the most optimal binding mode. The models for the simulation were generated with AlphaFold2 or downloaded from the PDB databank and prepared with the Protein Preparation Wizard, Schrödinger, LLC, New York^73^. The preparation adds the missing hydrogens, corrects the protonation states, fills the missing side chains and loops as wells as the disulfide bonds. Then the structures were manually checked for possible inconsistencies and were set up for the site finder protocol using 100 processors, 12 PELE steps and 50 iterations in a total of 60,000 Monte Carlos steps. Note that to make things equal, the Mg^2+^ ion was removed from the PDB structure of the ATPase which reduced the interaction energy with ATP. The simulation results with the Mg^2+^ is similar but the energy minimum close to the lysine reaches below − 100 kcal/mol.

## Supplementary Information

Supplementary Information.

## Data Availability

The datasets used and/or analysed during the current study are available in the FigShare repository, 10.6084/m9.figshare.22709599, and available from the corresponding author on reasonable request.
