# Supplementary material for: Insights into peculiar fungal LPMO family members holding a short C-terminal sequence reminiscent of phosphate binding motifs
Source: Sci Rep. 2023 Jul 18;13:11586. doi: 10.1038/s41598-023-38617-5 (PMC10354095; doi:10.1038/s41598-023-38617-5)
Supplement: Supplementary file 1 — Supplementary Information. [file 41598_2023_38617_MOESM1_ESM.pdf]

SUPPORTING INFORMATION FOR

Insights into peculiar fungal LPMO family members holding a short C-terminal sequence reminiscent of phosphate binding motifs

Jean-Lou Reyre[1,2], Sacha Grisel[1,6], Mireille Haon[1,6], Ruite Xiang[3], Jean-Charles Gaillard[5], Jean Armengaud[5], Victor Guallar[3,4], Antoine Margeot[2], Simon Arragain[2], Jean-Guy Berrin[1,6*] and Bastien Bissaro[1*]

[1]INRAE, Aix Marseille University, UMR1163 Biodiversité et Biotechnologie Fongiques, 13009 Marseille, France.
[2]IFP Energies nouvelles, 1 et 4 avenue de Bois-Préau, 92852 Rueil-Malmaison, France
[3]Barcelona Supercomputing Center, Plaça Eusebi Güell, 1-3, E-08034 Barcelona, Spain
[4]ICREA, Passeig Lluís Companys 23, E-08010 Barcelona, Spain
[5]Université Paris-Saclay, CEA, INRAE, Département Médicaments et Technologies pour la Santé (DMTS), SPI, F-30200, Bagnols-sur-Cèze, France.
[6]INRAE, Aix Marseille University, 3PE platform, 13009 Marseille, France.

*Corresponding authors:

Jean-Guy Berrin (jean-guy.berrin@inrae.fr)

Bastien Bissaro (bastien.bissaro@inrae.fr)

This file includes:

1. Supplementary Figure 1 to 11 and Supplementary table 1
2. Full abbreviations list
3. Supplementary references list

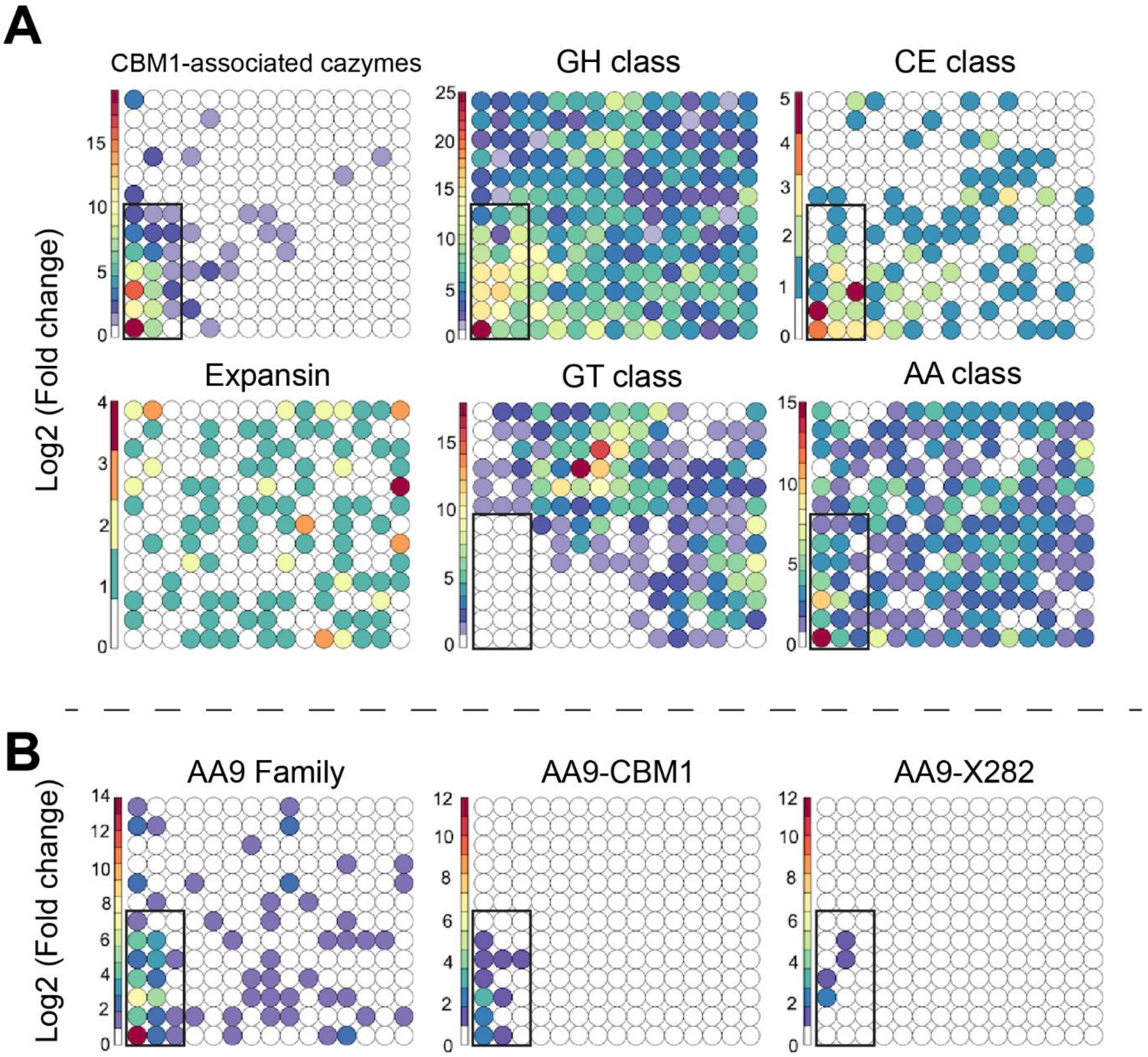

Fig. S1. Overview of the regulation of genes coding for predicted secreted proteins (including CAZymes) from six Polyporales species. The figure is adapted from Hage et al. 2021[1]. 5536 genes from six Polyporales species (*Artolenzites elegans*, *Leiotrametes sp.*, *Pycnoporus cinnabarinus*, *Pycnoporus coccineus*, *Trametes ljubarskyi* and *Irpex lacteus*) were grouped on the basis of their regulation similarity using Self-Organizing Map[2] and the resulting clusters were mapped into nodes (here 195), which locate in the vicinity of each other when sharing similar regulations patterns. The Tatami maps show, for each node, the mean log2 differential transcription (Blue: moderately transcribed, Yellow: over-transcribed, Red: highly transcribed; the log-color scale is provided in the figure) of genes at day 3 on cellulose (Avi), aspen (Asp), pine (Pin), and wheat straw (Whs) (merged data for the 4 lignocellulosic substrates) in comparison to maltose (reference condition). **(A)** Tatami maps showing for each node the counts of commonly up-regulated genes in response to cellulose, aspen, pine and wheat straw corresponding to: CBM1-associated CAZymes and glycoside hydrolase (GH), carbohydrate esterase (CE), expansin, glycosyl transferase (GT) and Auxiliary Activities (AA) classes. **(B)** Same maps as in panel A focusing on AA9, AA9-CBM1 and AA9-X282 genes. The cluster encompassing most of the cellulose-responsive genes are squared in black.

A

		Clade X1	Clade X2	Clade 1	Clade 2	Clade 3	Clade 4	Clade 5	Clade 6	Clade 7	Clade 8	Clade 9	Clade 10	Clade 11	Clade 12	Clade 13
Clade X1	Mean	76	57	44	38	36	32	35	37	34	37	36	33	39	43	43
Clade X2	Mean	57	78	43	36	38	31	30	34	32	29	33	29	38	43	43

B

		Clade X1	Clade X2	Clade 1	Clade 2	Clade 3	Clade 4	Clade 5	Clade 6	Clade 7	Clade 8	Clade 9	Clade 10	Clade 11	Clade 12	Clade 13
Clade X1	Min	57	48	38	31	28	28	31	33	29	32	30	29	33	37	36
	Max	100	66	49	42	41	39	42	41	40	41	41	38	43	47	48
Clade X2	Min	48	69	41	34	34	28	26	32	27	25	27	25	34	40	40
	Max	66	100	47	38	40	35	34	40	35	34	39	34	43	48	47

Fig. S2. Condensed percentage identity matrix. Percentage identity matrix of the sequences used to infer the phylogenetic analysis displayed in **Fig.1**. The clades are annotated from 1 to 13, the first one starting right after clade X2 in counterclockwise direction. Minimum, maximum, and average sequences percentage identity are displayed for each clade-to-clade comparison.

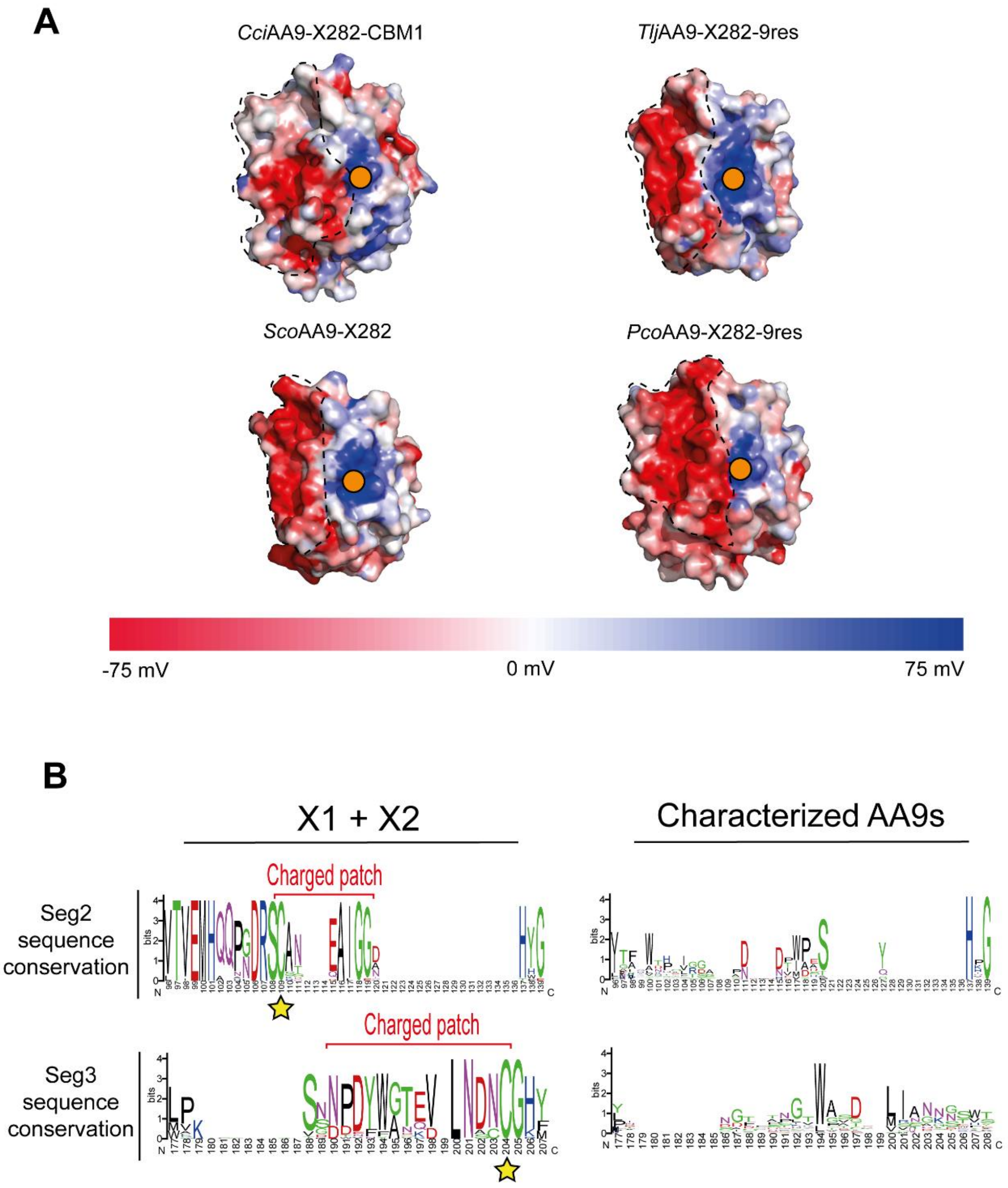

Fig. S3. Surface distribution of electrostatic potential (A) and presence of a conserved charged patch (B) in AA9-X282s. **(A)** Catalytic site view of four AA9-X282s (structural model predicted with AlphaFold2). Charge distributions were predicted using the ProtSol server[3] and represented as color gradient on the structure. The conserved negatively charged patch is circled with a black dotted line and the histidine-brace position is marked by an orange dot. **(B)** Residues conservation comparison of segments seg2 and seg3 (as defined by Laurent et al.[4]) between clades X1 (20 sequences) and X2 (4 sequences) *versus* the characterized AA9s (39 sequences). Residues of the charged patch are framed in red bracket on the Weblogos and the two strictly conserved cysteines from the charged patch are marked by a yellow star.

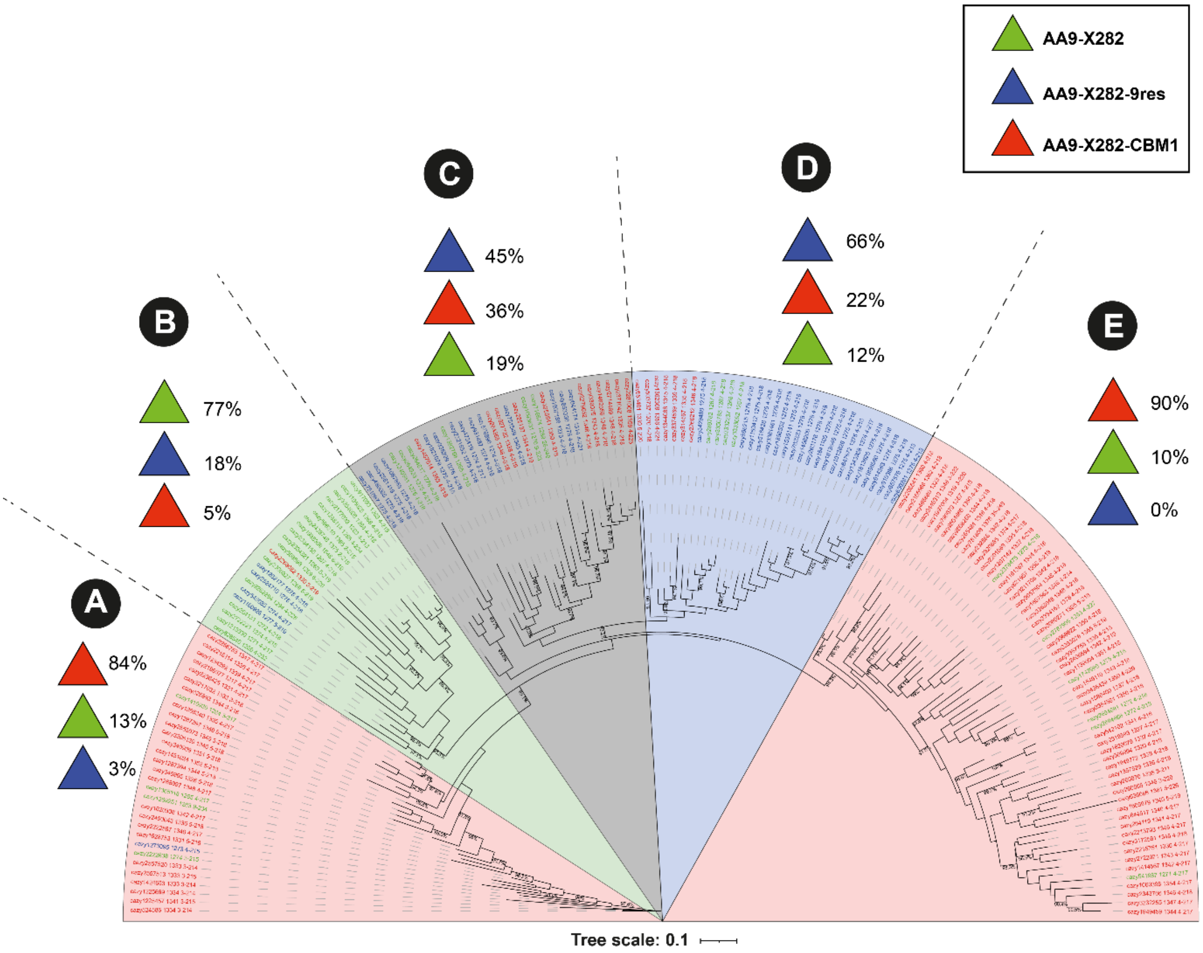

Fig. S4. Phylogeny and structural modularity of AA9-X282. Phylogenetic analysis of 174 AA9-X282 catalytic domains, showing 5 clades (A to E). For each clade, the associated proportions of the three AA9-X282 modularities are shown on the tree. Color code: AA9-X282 in green, AA9-X282-CBM1 in red and AA9-X282-9res in blue. The tree was generated with IQ-Tree (1000 Bootstraps). The protein IDs are CAZy IDs and the corresponding sequences are available upon request.

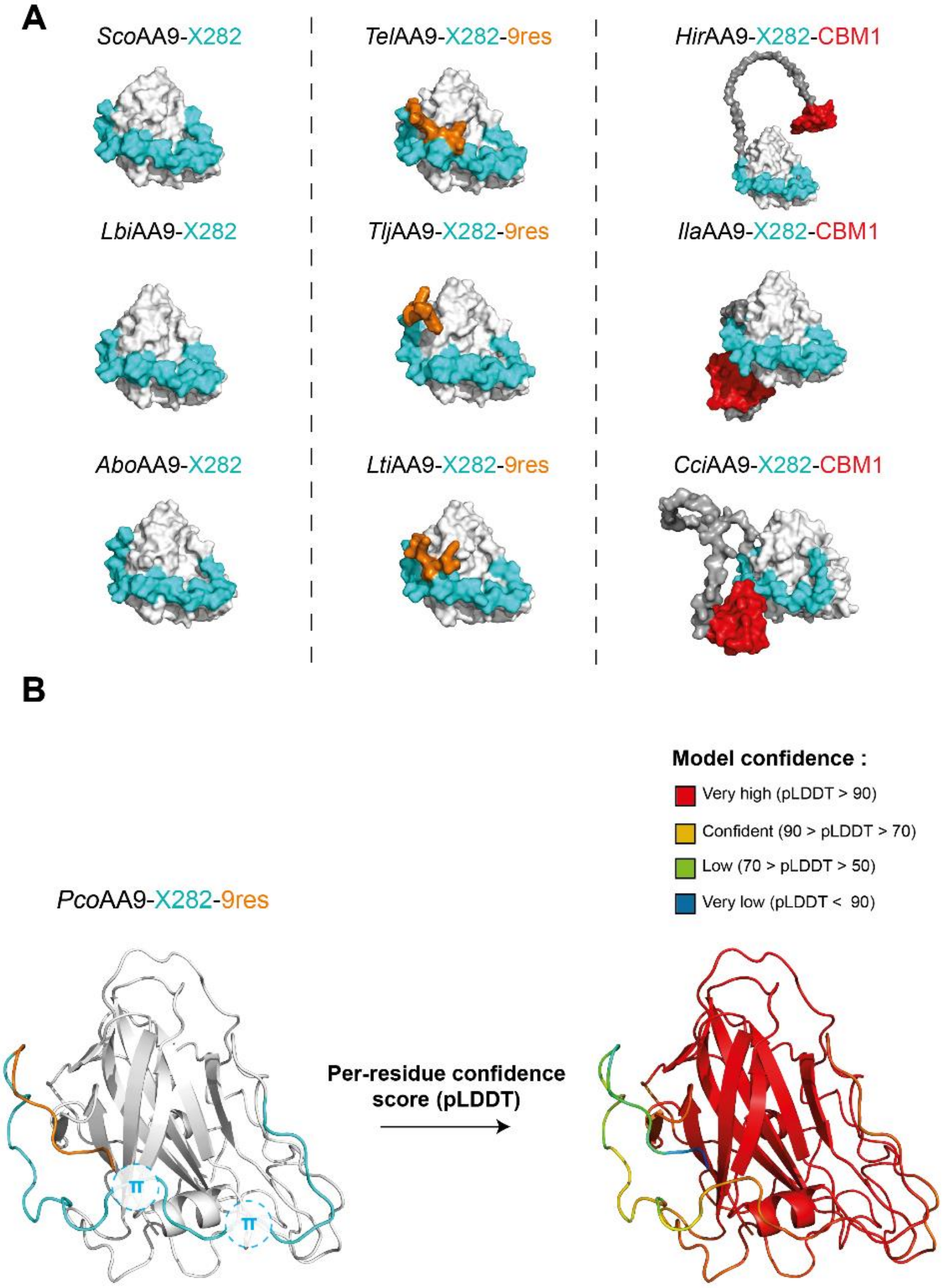

Fig. S5. Structure predictions of 10 AA9-X282s. (A) Structures, predicted with AlphaFold2[5], for AA9-X282s from *Schizophyllum commune* (*Sco*), *Laccaria bicolor* (*Lbi*), *Armillaria boreal* (*Abo*); AA9-X282-9res from *Trametes elegans* (*Tel*), *Trametes ljubarskyi* (*Tlj*), *Lentinus tigrinus* (*Lti*); AA9-X282-CBM1 from *Heterobasidion irregulare* (*Hir*), *Irpex lacteus* (*Ila*) and *Coprinopsis cinerea* (*Cci*). The X282 (in cyan), the 9res motif (in orange), the linker (in dark grey) and the CBM1 (in red) are colored on the structures shown as surface. **(B)** Per-residue confidence score (pLDDT) of *Pco*AA9-X282-9res structure prediction provided by Alphafold2[5]. The color code corresponding to the model confidence is displayed on the figure and the position of the two potential π-stacking interactions are indicated on *Pco*AA9-X282-9res structure prediction.

A

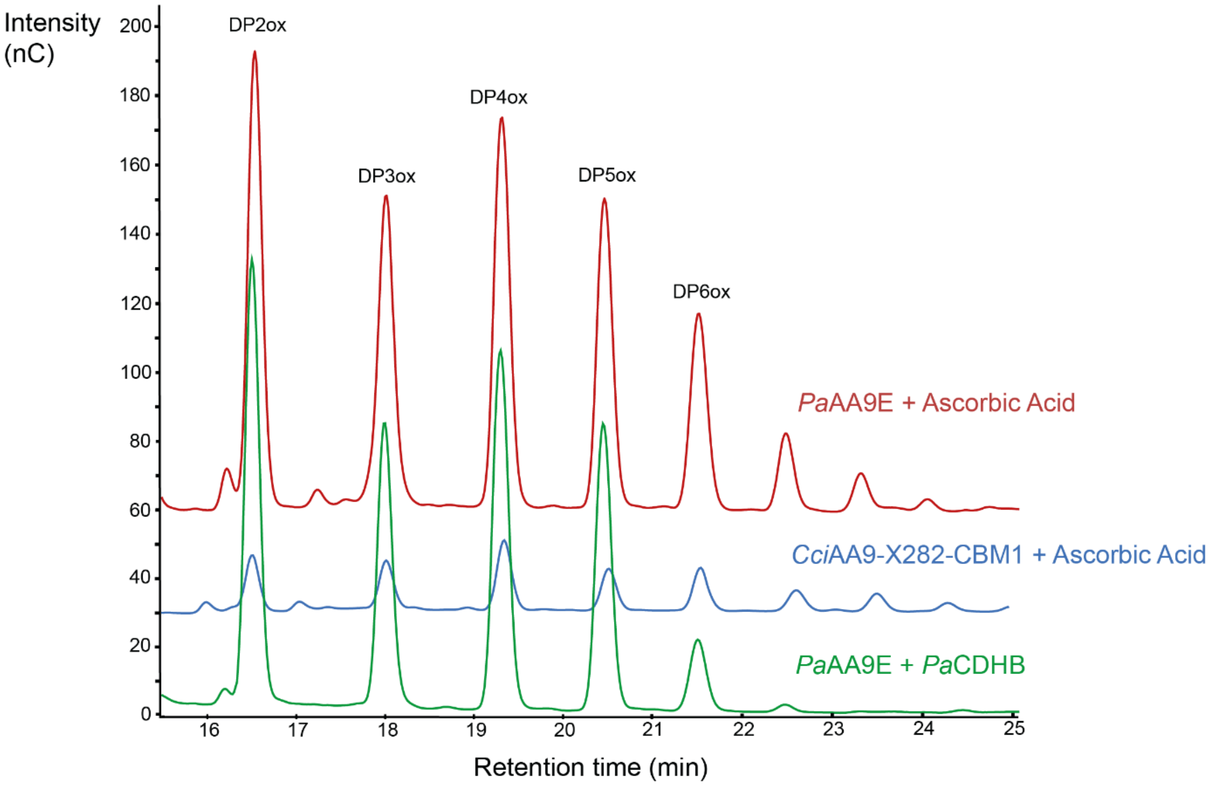

B

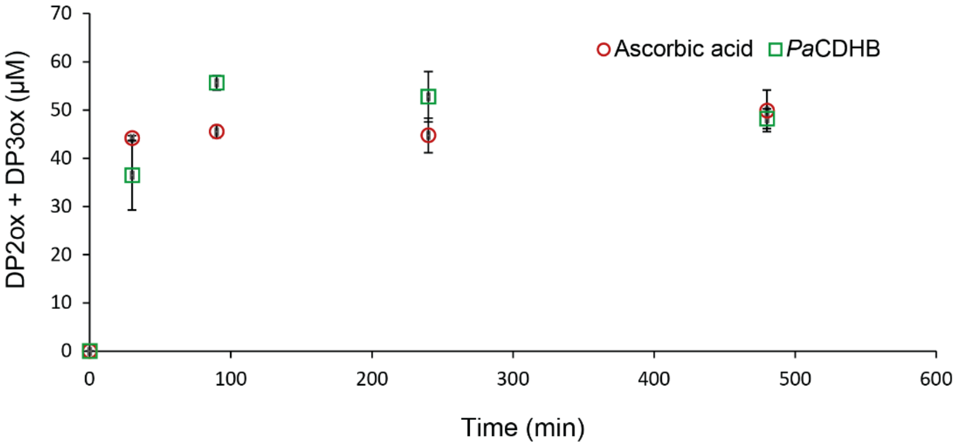

Fig. S6. Comparative kinetic of *Pa*AA9E reactions on PASC in the presence of *Pa*CDHB or ascorbic acid. (A) Oxidized cello-oligosaccharides (DP2-DP6ox) released by *Pa*AA9E (1 µM) from PASC (0.2%) after 90 min of reaction, in the presence of ascorbic acid (1 mM) or *Pa*CDHB (1 µM). The oxidized products released by *Cci*AA9-X282-CBM1 (1 µM) after 24 h reaction in the presence ascorbic acid (1 mM) is also displayed for comparison. Reactions were carried out in sodium acetate buffer (50 mM, pH 5.2) and incubated in a Thermomixer (850 rpm, 30°C). **(B)** Time course kinetic of the released C1-oxidized soluble products (DP2ox and DP3ox) from the *Pa*AA9E reactions described in **(A).** Data points show average values and error bars show standard deviations (n = 3 independent biological replicates).

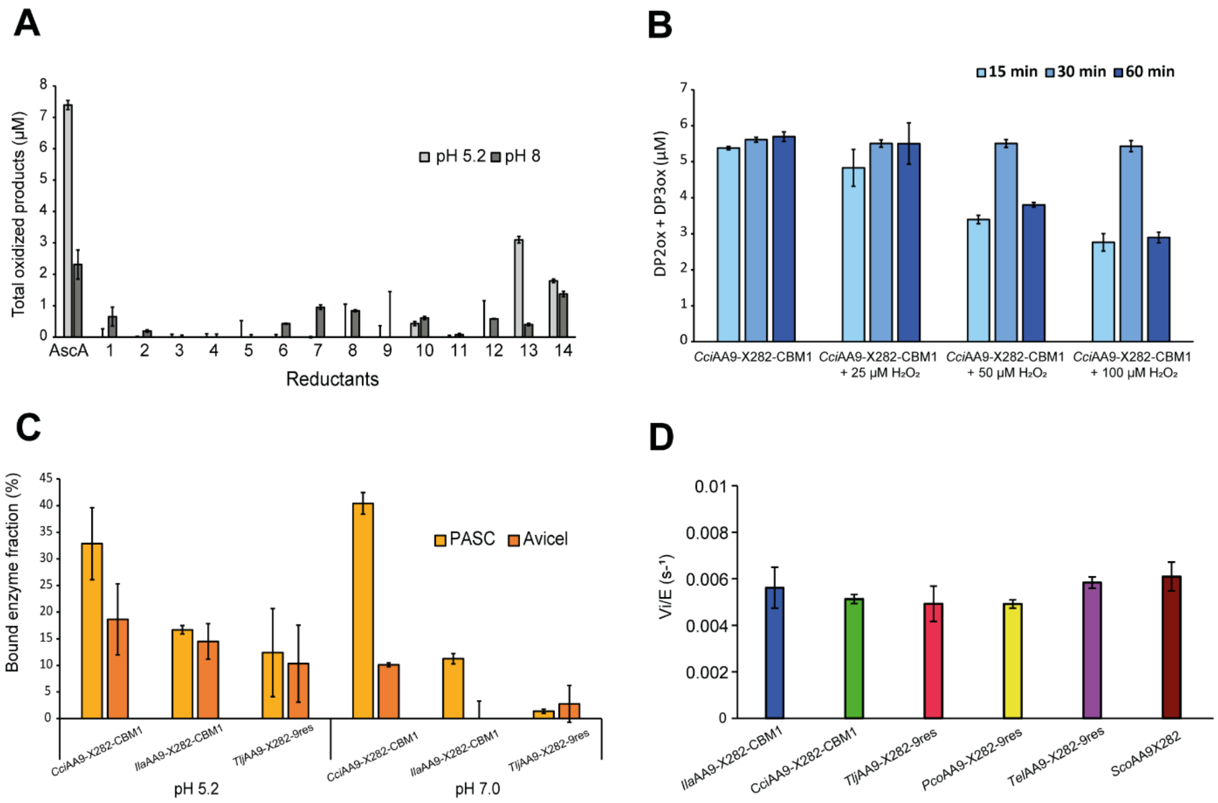

Fig. S7. Biochemical and binding assays of AA9-X282s. (A) C1-oxidized cello-oligosaccharides released from PASC by *Ila*AA9-X282-CBM1 (1 µM) in the presence of different lignin-derived compounds as reductants (see list with full names corresponding to numerical code in **Table S1**). **(B)** Effect of H_2O_2 complementation on the time-course release of oxidized products by *Cci*AA9-X282-CBM1 (1 µM) from PASC (0.2%), in the presence of AscA (1 mM). **(C)** Binding assay on PASC (0.2%) and Avicel (5 mg.mL^{-1}) for *Cci*AA9-X282-CBM1, *Ila*AA9-X282-CBM1 and *Tlj*AA9-X282-9res (at 10 µM each). **(D)** Oxidase activity of the six AA9-X282s (1 µM) assayed with Amplex-red. Histograms show average values and error bars show standard deviations (n = 3 independent biological replicates).

A

*Pco*AA9-X282-9res
Catalytic domain

Position and residue in *Pco* AA9-X282-9res	Phosphorylated in (legend squares)	Serine conservation in AA9-X282 (%)	Threonine conservation in AA9-X282 (%)
Thr3	*Cci*, *Tlj*, *Pco*	0	99
Ser16		65	5
Ser17		57	3
Ser24		93	0
Ser26	*Pco*	75	0
Thr29		1	89
Ser30		64	0
Thr32	*Pco*	17	75
Thr33	*Pco*	59	35
Ser41	*Cci*, *Pco*	8	5
Ser45		85	4
Thr58		5	94
Ser69		90	6
Thr72		5	13
Thr94		4	70
Thr95		26	73
Ser99		72	3
Ser100	*Tlj*	65	2
Ser113	*Pco*	99	0
Ser114	*Tlj*	29	1
Thr121		4	76
Thr132		2	94
Ser137		38	12
Ser157		83	0
Ser158		66	13
Ser178		44	53
Thr183		0	81
Ser185		34	1
Ser191		66	0
Thr193		29	29
Ser206		18	22
Thr207		18	53
Thr209		1	37
Thr214		20	58
Thr218		11	69
Thr219		10	54

*Pco*AA9-X282-9res
C-term

Position and residue in *Pco* AA9-X282-9res	Serine conservation in AA9-X282 (%)	Threonine conservation in AA9-X282 (%)
Ser220	71	0
Thr226	3	94
Thr230	2	89
Thr231	3	84
Thr233	1	99
Thr236	0	99
Ser241	66	17
Thr242	4	89
Thr245	4	88
Thr249	8	29
Thr252	11	21

Phosphorylated in...
- ...*Pco*AA9-X282-9res (light blue)
- ...*Tlj*AA9-X282-9res (dark blue)
- ...*Cci*AA9-X282-CBM1 (red)

B

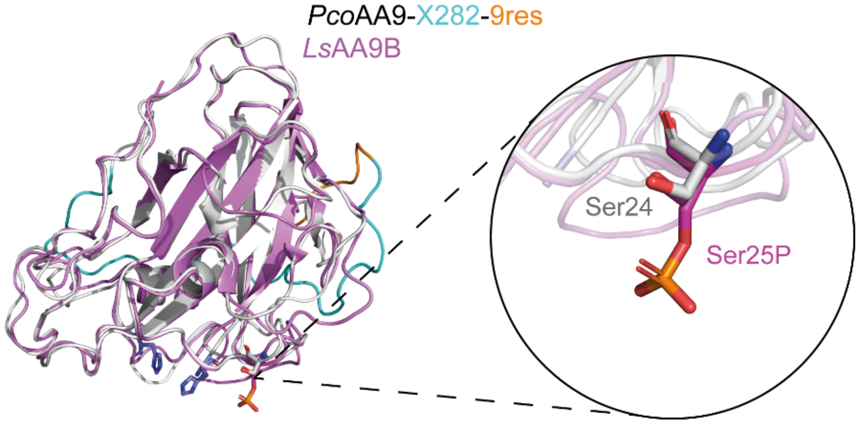

Figure S8. Conservation of surface exposed serines and threonines in AA9-X282. (A) *Pco*AA9-X282-9res serine and threonine conservation rate throughout 174 AA9-X282 sequences. Phosphorylated residues in *Pco*AA9-X282-9res and their structural equivalents in *Tlj*AA9-X282-9res or *Cci*AA9-X282-CBM1 are annotated by colored squares (note that the Ser41 phosphorylated in *Cci*AA9-X282-CBM1 had no Ser/Thr equivalent in *Pco*AA9-X282-9res and is thus not represented). **(B)** Structural alignment of *Pco*AA9-X282-9res (in white) with *Ls*AA9B (in purple). A zoom-in on the protruding phosphorylated Ser25 in *Ls*AA9B and its equivalent in *Pco*AA9-X282-9res is circled in black. The two catalytic histidines are colored in blue.

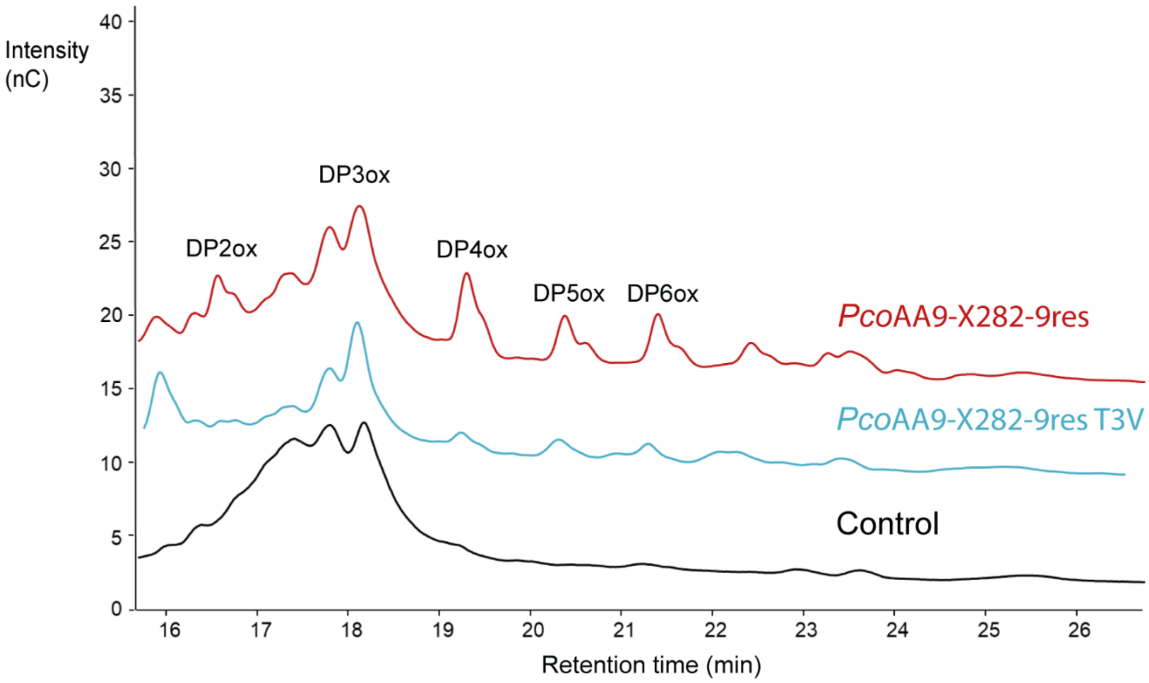

Fig. S9. Soluble oxidized products profile of *Pco*AA9-X282-9res and T3V variant. HPAEC-PAD chromatogram of soluble oxidized products released from PASC (0.2%) by *Pco*AA9-X282-9res (1 µM) bearing either the original Thr at position 3 (*Pco*AA9-X282-9res, in red) or the T3V mutation (*Pco*AA9-X282-9res T3V, in blue), in the presence of ascorbic acid (1 mM). Reactions were incubated in sodium acetate buffer (50 mM, pH 5.2), during 8 h, under stirring (850 rpm) at 30°C. The control reaction was made by replacing the AA9 by $CuSO_4$ (1 µM).

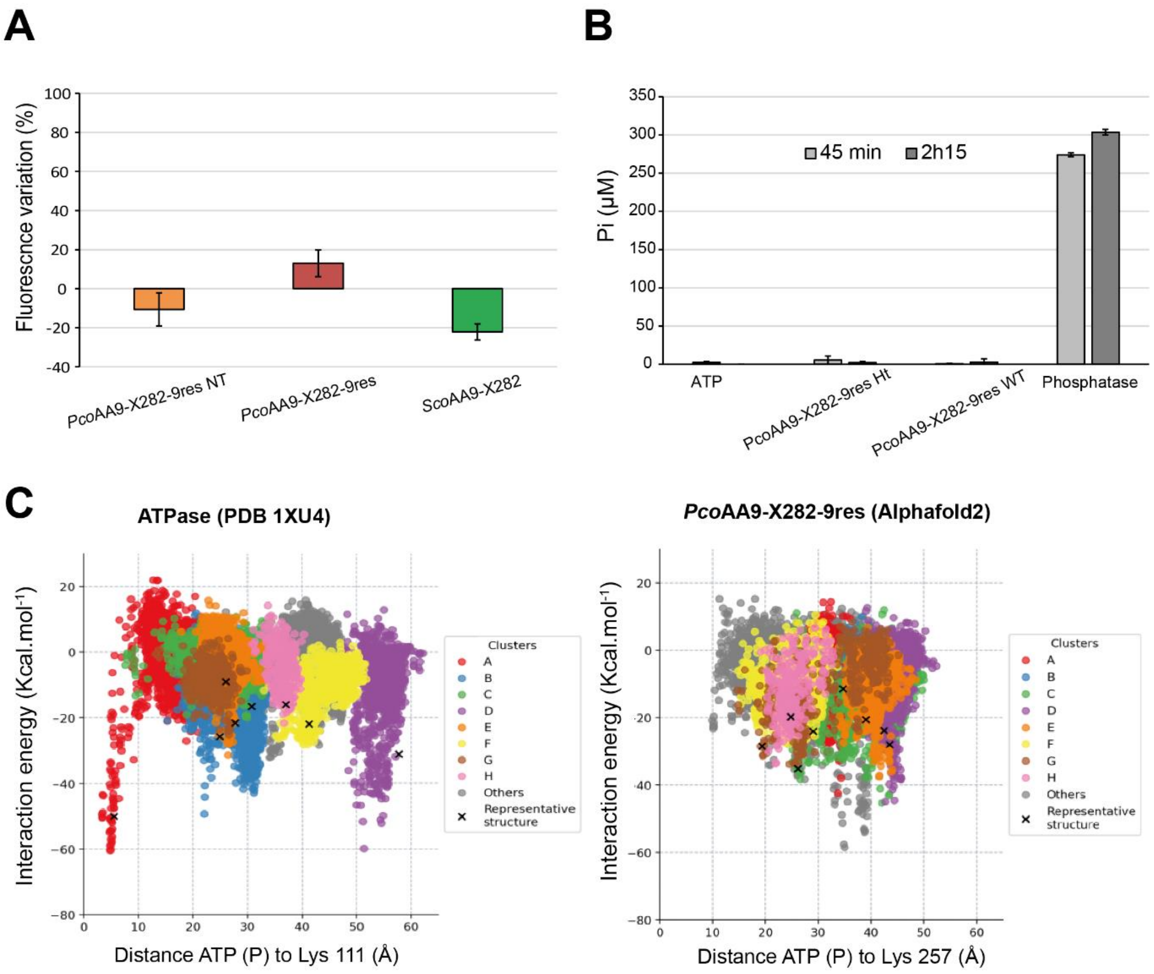

Fig. S10. ATP binding assays. (A) TNP-ATP assay: TNP-ATP (20 µM) was mixed with*Pco*AA9-X282-9res NT (in orange), *Pco*AA9-X282-9res (in red) or *Sco*AA9-X282 (in green) (10 µM for each protein). After 5 min, the fluorescence emission was read at 540 nm upon excitation at 410 nm. Control experiments consisted in recording the fluorescence emission background signal of TNP-ATP and AA9 proteins alone. All binding assays were performed in Tris-HCl buffer (50 mM, pH 7.5). When a genuine ATP-binding protein binds to TNP-ATP, the fluorescence of the latter is expected to increase significantly, usually >2-fold[6]. The variation in fluorescence (F) shown on the y-axis corresponds to the variation of F(protein + TNP-ATP) relative to the sum of F(protein) and F(TNP-ATP). y = 0% means that addition of protein to TNP-ATP has no effect on the fluorescence of the latter, reflecting thereby the absence of binding. **(B)** ATP hydrolysis assay: Released inorganic phosphate (Pi) in complex with the malachite green reagent was monitored at 640 nm. Reactions were performed by mixing AA9-X282 (1 µM) or commercial acidic phosphatase (100 nM) with ATP (200 µM). Reactions were performed in sodium acetate buffer (50 mM, pH 5.2) in the presence of $MgCl_2$ (100 µM). **(C)** Site finder simulation. The simulation was performed using an AlphaFold2 model of the *Pco*AA9-X282-9res to detect possible binding sites for ATP, and the results were compared to an ATPase (PDB: 1XU4) as the control. Each point is a different conformation of the binary complex between the ATP and the receptor. The distance between the γ-phosphate and the lysine of the "GXXGIGKA" consensus sequence, in the case of the *Pco*AA9, or the Walker A motif, in the case of the ATPase, is plotted against the interaction energies between the ligand and the protein. The colors show different clusters grouped by ligand RMSD as reported by PELE.

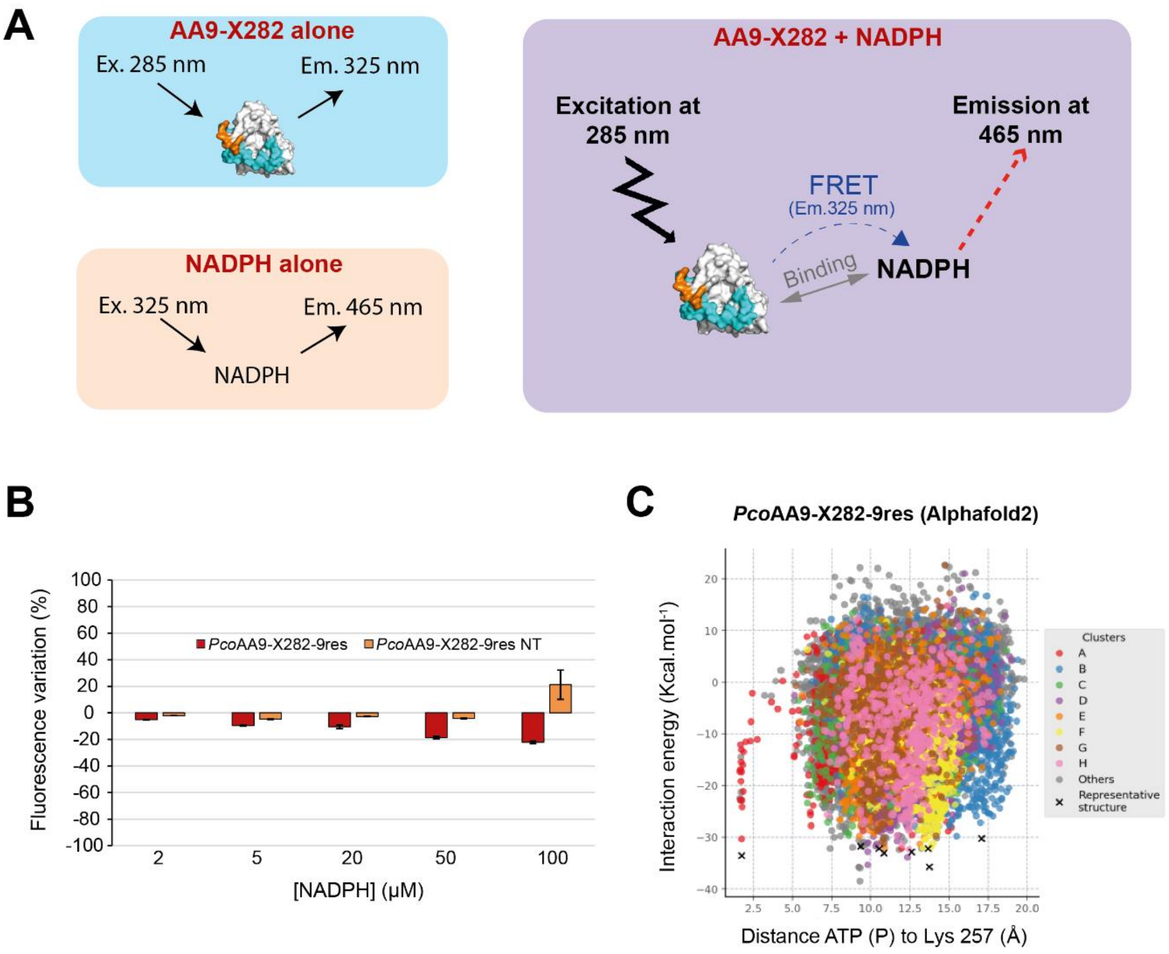

Fig. S11. NADPH binding assays. (A) Summary scheme of the method. The NADPH binding assay is based on the method described in Fjeld et al.[7]. When the protein is excited at 285 nm, its emission at 325 nm will be quenched by NADPH via a FRET mechanism if the NADPH is bound to the protein, leading to a specific final emission at 465 nm. **(B)** Evolution of the fluorescence emission (at 325 nm) upon excitation at 285 nm of the *apo-Pco*AA9-X282-9res (5 µM), with and without ("NT") His-tag, in the presence of different concentrations of NADPH. The variation in fluorescence (F) shown on the y-axis corresponds to the variation of F(protein + NADPH) relative to the F(protein). **(C)** Site finder simulations. The Protein-NADPH interaction energy was plotted as a function of the distance between the protein (the Gly256, next to the Lys in the "GXXGIGKA" consensus sequence) and NADPH (one of the oxygens in the phosphodiester bond). The coloured clusters indicate different conformations of NADPH group by RMSD. Each plot point corresponds to a different binary complex obtained during the PELE site finder simulation. We note that for canonical NAD(P)H binding proteins, one may expect interaction energies in the range -100 to -140 kcal/mol).

Table S1. List of the screened substrates and reductants

Tested reductants	Number in Fig. S6A
Ascorbic acid	*/*
4-hydrobenzoic acid	1
p-coumaric acid	2
Vanillic acid	3
Vanillin	4
Isovanillin	5
Eugenol	6
4-ethyl-guaiacol	7
Ferulic acid	8
Homovanillyl alcohol	9
2,6-dimethoxyphenol	10
Syringaldehyde	11
Sinapic acid	12
2-methoxyhydroquinone	13
Caffeic acid	14

Tested substrates	Detected activity
PASC	Low
Avicel	Very low
Cello-oligosaccharides (DP2-DP6)	No activity
PASC/Xylan coupling	
Xyloglucan	
Xylan (from beechwood)	
Xylo-oligosaccharides	
Glucorono xylan	
Arabinoxylan	
Arabinan	
Glucomannan	
Galactomannan	
Pectic galactan	
Rhamnogalacturonan	
α-Chitin	
β-Chitin	

Full abbreviations list

Enzymes/Microorganisms

AA: Auxiliary activities
HRP: Horseradish peroxidase
Pco: Pycnoporus cinnabarinus
Tlj: *Trametes ljubarskyi*
Tel: Trametes elegans
Cci: Coprinopsis cinerea
Ila: Irpex lacteus
Sco: Schizophyllum commune

Substrates/products

PASC: Phosphoric acid swollen cellulose
TNP-ATP: 2',3'-O-Trinitrophenyl-ATP
PCW: Plant cell wall
YPD: Yeast Extract–Peptone–Dextrose
BMGY: Buffered Glycerol- complex Medium
BMMY: Buffered Methanol- complex Medium

Methods

HPAEC-PAD: High-performance anion-exchange chromatography coupled with pulsed amperometric detection
MS/MS: Tandem mass spectrometry
UHPLC: Ultra High-Performance Liquid Chromatography
PELE: Protein energy landscape exploration
MSA: Multiple sequences alignment
MW: Molecular weight

References

1. Hage, H. *et al.* Gene family expansions and transcriptome signatures uncover fungal adaptations to wood decay. *Environmental Microbiology* **23,** 5716–5732 (2021).
2. Miyauchi, S. *et al.* Conserved white-rot enzymatic mechanism for wood decay in the Basidiomycota genus Pycnoporus. *DNA Res* **27** (2020).
3. Hebditch, M., Carballo-Amador, M. A., Charonis, S., Curtis, R. & Warwicker, J. Protein-Sol: a web tool for predicting protein solubility from sequence. *Bioinformatics* **33,** 3098–3100 (2017).
4. Laurent, C. V. F. P. *et al.* Influence of Lytic Polysaccharide Monooxygenase Active Site Segments on Activity and Affinity. *International journal of molecular sciences* **20,** 6219 (2019).
5. Jumper, J. *et al.* Highly accurate protein structure prediction with AlphaFold. *Nature* **596,** 583–589 (2021).
6. Hiratsuka, T. Fluorescent and colored trinitrophenylated analogs of ATP and GTP. *European journal of biochemistry* **270,** 3479–3485 (2003).
7. Fjeld, C. C., Birdsong, W. T. & Goodman, R. H. Differential binding of NAD+ and NADH allows the transcriptional corepressor carboxyl-terminal binding protein to serve as a metabolic sensor. *Proceedings of the National Academy of Sciences of the United States of America* **100,** 9202–9207 (2003).
